# Loss of the interferon-γ-inducible regulatory immunity-related GTPase (IRG), Irgm1, causes activation of effector IRG proteins on lysosomes, damaging lysosomal function and predicting the dramatic susceptibility of Irgm1-deficient mice to infection

**DOI:** 10.1186/s12915-016-0255-4

**Published:** 2016-04-20

**Authors:** Jelena Maric-Biresev, Julia P. Hunn, Oleg Krut, J. Bernd Helms, Sascha Martens, Jonathan C. Howard

**Affiliations:** Institute for Genetics, University of Cologne, Cologne, Germany; Department for Biochemistry and Cell Biology, Faculty of Veterinary Medicine and Institute of Biomembranes, Utrecht University, Utrecht, The Netherlands; Institute for Medical Microbiology, Immunology and Hygiene, University Hospital Cologne, Cologne, Germany; Max F. Perutz Laboratories, University of Vienna, Vienna, Austria; Instituto Gulbenkian de Ciência, Rua da Quinta Grande 6, Oeiras, Portugal; Max-Planck Institute for Plant Breeding Research, Cologne, Germany

**Keywords:** Immunity-related GTPases, IRGs, Pathogen recognition, Irgm1, Irgm3, Lysosomes, Autophagy

## Abstract

**Background:**

The interferon-γ (IFN-γ)-inducible immunity-related GTPase (IRG), Irgm1, plays an essential role in restraining activation of the IRG pathogen resistance system. However, the loss of Irgm1 in mice also causes a dramatic but unexplained susceptibility phenotype upon infection with a variety of pathogens, including many not normally controlled by the IRG system. This phenotype is associated with lymphopenia, hemopoietic collapse, and death of the mouse.

**Results:**

We show that the three regulatory IRG proteins (GMS sub-family), including Irgm1, each of which localizes to distinct sets of endocellular membranes, play an important role during the cellular response to IFN-γ, each protecting specific membranes from off-target activation of effector IRG proteins (GKS sub-family). In the absence of Irgm1, which is localized mainly at lysosomal and Golgi membranes, activated GKS proteins load onto lysosomes, and are associated with reduced lysosomal acidity and failure to process autophagosomes. Another GMS protein, Irgm3, is localized to endoplasmic reticulum (ER) membranes; in the Irgm3-deficient mouse, activated GKS proteins are found at the ER. The Irgm3-deficient mouse does not show the drastic phenotype of the Irgm1 mouse. In the Irgm1/Irgm3 double knock-out mouse, activated GKS proteins associate with lipid droplets, but not with lysosomes, and the *Irgm1/Irgm3*^*−/−*^ does not have the generalized immunodeficiency phenotype expected from its Irgm1 deficiency.

**Conclusions:**

The membrane targeting properties of the three GMS proteins to specific endocellular membranes prevent accumulation of activated GKS protein effectors on the corresponding membranes and thus enable GKS proteins to distinguish organellar cellular membranes from the membranes of pathogen vacuoles. Our data suggest that the generalized lymphomyeloid collapse that occurs in *Irgm1*^*−/−*^ mice upon infection with a variety of pathogens may be due to lysosomal damage caused by off-target activation of GKS proteins on lysosomal membranes and consequent failure of autophagosomal processing.

**Electronic supplementary material:**

The online version of this article (doi:10.1186/s12915-016-0255-4) contains supplementary material, which is available to authorized users.

## Background

The identification and eradication of intracellular parasites that are enclosed in host-derived vacuolar membranes poses the question, how are these new structures to be distinguished from endogenous membrane-bound cytoplasmic organelles? The issue is especially acute for organisms such as *Toxoplasma gondii*, which do not enter cells via the phagosomal route, but rather by an active entry mechanism independent of all host uptake machinery. In mice, resistance to such pathogens is dependent on the interferon-γ (IFN-γ)-inducible system of immunity-related GTPases (IRG proteins) [1, 2]. The effector proteins of this system rapidly accumulate and activate at the vacuolar membranes of a disparate group of intracellular parasites, namely the protozoan *T. gondii* [3–9], the bacterium *Chlamydia trachomatis* [10–13], and the microsporidian fungus *Encephalitozoon cuniculi* [14], but not at the membranes of many other intracellular organisms. The known target organisms all share the property of entering host cells by non-phagocytic mechanisms. The accumulation of activated IRG proteins on the cytosolic face specifically of parasitophorous vacuole membranes (PVMs) seems to imply that these membrane-bound structures are distinct from endogenous membrane-bound intracellular compartments, but the mechanism by which IRG proteins activate only on pathogen-containing vacuoles is not fully understood.

**Fig. 1:**
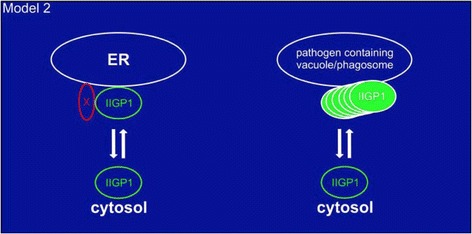
Oligomerization model of Irga6 proposed by Martens in 2004 [15]. Irga6 (labelled according to the old nomenclature as IIGP1) shuttles between endoplasmic reticulum membranes and cytosol. Nucleotide-dependent oligomerization of Irga6 is prevented at the membrane by a yet unknown factor (X). X is missing from the *Toxoplasma gondii* parasitophorous vacuole allowing Irga6 oligomerization at the vacuole

In 2004, Martens [15] hypothesized that activation at endogenous membranes is inhibited by the presence of negative regulatory self-proteins (designated X) that block the activation of IRG proteins on these membranes (Fig. 1).

In this proposal, X proteins are missing on newly formed pathogen-containing vacuoles, such as those of *T. gondii*, thus allowing IRG proteins to activate and concentrate on this compartment. We subsequently showed [16] that a structurally distinct subset of three IRG proteins, the IRGM or GMS subset, fulfill the definition of X. The GMS subset are restricted to specific organellar compartments [17, 18], they inhibit the activation of the effector (GKS) subset of IRG proteins by blocking nucleotide exchange, and they are either absent or very weakly expressed on *T. gondii* PVMs [19]. In their absence, effector GKS proteins activate spontaneously in the cytoplasm. This model has been reiterated in subsequent publications from our laboratory [20], and recently restated as “missing self” from another laboratory [21, 22].

The GMS proteins are tightly associated with distinct compartments of the cellular endomembrane system. In uninfected cells, Irgm1 localizes strongly to the Golgi apparatus [17, 23, 24] but also to the endolysosomal compartment [23, 25], mitochondria [24, 26, 27], peroxisomes [21, 24], and to lipid droplets [21]. Irgm1 is also found on phagocytic cups containing latex beads and on sterile phagosomes containing ferritin and latex beads [17, 23, 25]. However, contrary to earlier claims based on organelle purification [28] or transfected, tagged constructs [29, 30], Irgm1 is not detectably present on either listerial or mycobacterial phagosomes [27]. Irgm2 localizes to the Golgi [18] and Irgm3 to the endoplasmic reticulum (ER) [17, 31, 32] and lipid droplets [32] and has been reported on magnetically purified latex bead phagosomes [23]. In IFN-γ-induced wild type (WT) cells, the effector (GKS) IRG proteins are predominantly cytosolic and in the inactive GDP-bound state [33]. All three GMS regulators are required for the control of GKS activation in the cell: when GKS proteins are expressed in the cell in the absence of one or more GMS proteins, they activate spontaneously, form aggregate-like structures, and do not accumulate on the *T. gondii* PVM [8, 16, 17].

Until now, disruptions of Irgm1 and Irgm3 have been described [3, 4]. Loss of Irgm3 results in a specific loss of function against just that subset of parasites listed above that seem to be the focus of the IRG resistance mechanism [3, 4]. Loss of Irgm1, on the other hand, has a drastic phenotype severely weakening mouse resistance to a number of pathogens, not only to *T. gondii*, *C. trachomatis*, and *E. cuniculi*, but also to pathogens that are not controlled by GKS proteins such as *Listeria* [4, 9], *Mycobacterium* [24, 28, 34, 35], *Salmonella* [6], *Leishmania* [36], and *Trypanosoma* [37]. *Irgm1*^*−/−*^ mice are also reported to be unusually susceptible to lipopolysaccharide injection [38], in the mouse model of colitis [39], in experimental immune encephalitis [40, 41], and in the mouse model of stroke [42]. Due to the complexity of this phenotype, very different and sometimes contradictory functions have been attributed to Irgm1 [20, 35, 43, 44]. In an infection setting, it is clear that Irgm1 cannot exert a direct effector action on phagosomes or PVMs because, despite earlier claims [24, 28–30], Irgm1 is not present on pathogen-containing phagosomal or vacuolar membranes [27]. Whatever action it performs in the control of infection must therefore be indirect. The immune defect in *Irgm1*^−/−^ mice appears to follow at least in part from a severe defect in their hematopoietic stem cells (HSCs), which lack renewal capacity [45]. Infection of *Irgm1*^−/−^ mice with any pathogen that stimulates IFN-γ induction, for instance *Salmonella typhimurium* [46] or *Mycobacterium avium* [34], results in a generalized lymphomyeloid collapse [35, 47]. Additionally, memory T cells from Irgm1-deficient mice have an IFN-γ-dependent proliferation defect following antigen restimulation and a large proportion of restimulated cells die [35]. In view of the severe phenotype of the Irgm1 mouse, it was therefore a great surprise when it was shown that the double mutant *Irgm1/Irgm3*^−/−^ mouse reverts back to the limited and precise phenotypic deficiency of the *Irgm3*^*−/−*^ single mutant [6]. Thus, the drastic defect of Irgm1 deficiency is somehow repaired by additional Irgm3 deficiency. This striking result further contradicts any direct anti-pathogenic effector model of Irgm1 action, leaving the severe cellular defects of the Irgm1-deficient mouse in need of an explanation that satisfies these paradoxical data.

Since GMS proteins maintain GKS proteins in the GDP-bound inactive state [16], we and others have proposed that GMS proteins protect cellular endomembranes from GKS action [20, 21], essentially according to the “missing X” model of Martens [15]. Following this idea, herein, we have concentrated on the striking fact that there are three different GMS proteins, each localized to specific organellar compartments in the IFN-γ-stimulated cell. Furthermore, all three GMS proteins are needed to prevent spontaneous activation of GKS proteins [16]. Since the cellular defects in Irgm1-deficient mice are dependent on IFN-γ signaling, we hypothesize here that the absence of specific GMS proteins should result in accumulation and activation of IFN-γ-induced GKS proteins on specific cytoplasmic organelles, and this might result in distinct targeted cellular pathologies characteristic of each GMS deficiency. Specifically, loss of Irgm1 could result in activation of GKS proteins on lysosomes, while loss of Irgm3 could result in activation of GKS proteins on ER membranes.

In the first description of the Irgm1 deficient mouse [4], the authors speculated that the protein might be involved in transport of materials to PVMs, and included a possible role in acidification of lysosomes. Subsequently, a defect in acidification was found in lysosomes from mycobacterial phagosomes in macrophages from Irgm1-deficient mice [28], and more recently evidence has been adduced for autophagic flux impairment in IFN-γ-induced Irgm1-deficient cells [48], consistent with a lesion associated with lysosomal function. In cells deficient for Irgm1 alone, we now show that IFN-γ-induced GKS proteins accumulate in the active state specifically on lysosomal membranes, while in *Irgm3*^−/−^ cells GKS proteins accumulate and activate specifically on ER membranes. As recently shown [21] and here confirmed, in *Irgm1/Irgm3*^−/−^ cells GKS proteins are, surprisingly, localized on lipid droplets, but not on lysosomes or ER as expected from the Irgm1 or Irgm3 deficiencies individually. In Irgm1-deficient cells we show that GKS-coated lysosomes have an acidification defect and their ability to process autophagosomes and other substrates is impaired. Thus, autophagic flux, which is essential for lymphocyte survival [49, 50], is partially blocked and may well be responsible for the lymphomyeloid defects and lymphopenia in *Irgm1*^−/−^ mice. In contrast, in IFN-γ-induced cells from *Irgm3*^−/−^ and *Irgm1/Irgm3*^−/−^ mice, which have no generalized immunodeficiency, GKS proteins do not accumulate on lysosomes and autophagic flux is normal. In view of the documented sensitivity of lymphomyeloid cells to autophagic injury [49, 50], we propose that the autophagic pathology induced by IFN-γ in Irgm1-deficient cells is responsible for the generalized immunodeficiency of the Irgm1 mouse. The shift of activated GKS proteins from lysosomes to lipid droplets in the Irgm1/Irgm3 double-deficient mouse is thus responsible for the unexpected rescue of Irgm1 immunodeficiency.

## Results

### Irga6 co-localizes with the lysosomal compartment in Irgm1-deficient cells

Irgm1 is the only GMS protein known to localize to the lysosomal compartment [25], while Irgm3 is the only GMS protein known to localize to the ER [31]. Therefore, if GMS proteins protect the endocellular membranes against GKS protein activation, Irgm1 deficiency should result in accumulation of activated GKS proteins on lysosomal membranes, while Irgm3 deficiency should result in accumulation of activated GKS proteins, such as Irga6, on ER membranes.

**Fig. 2:**
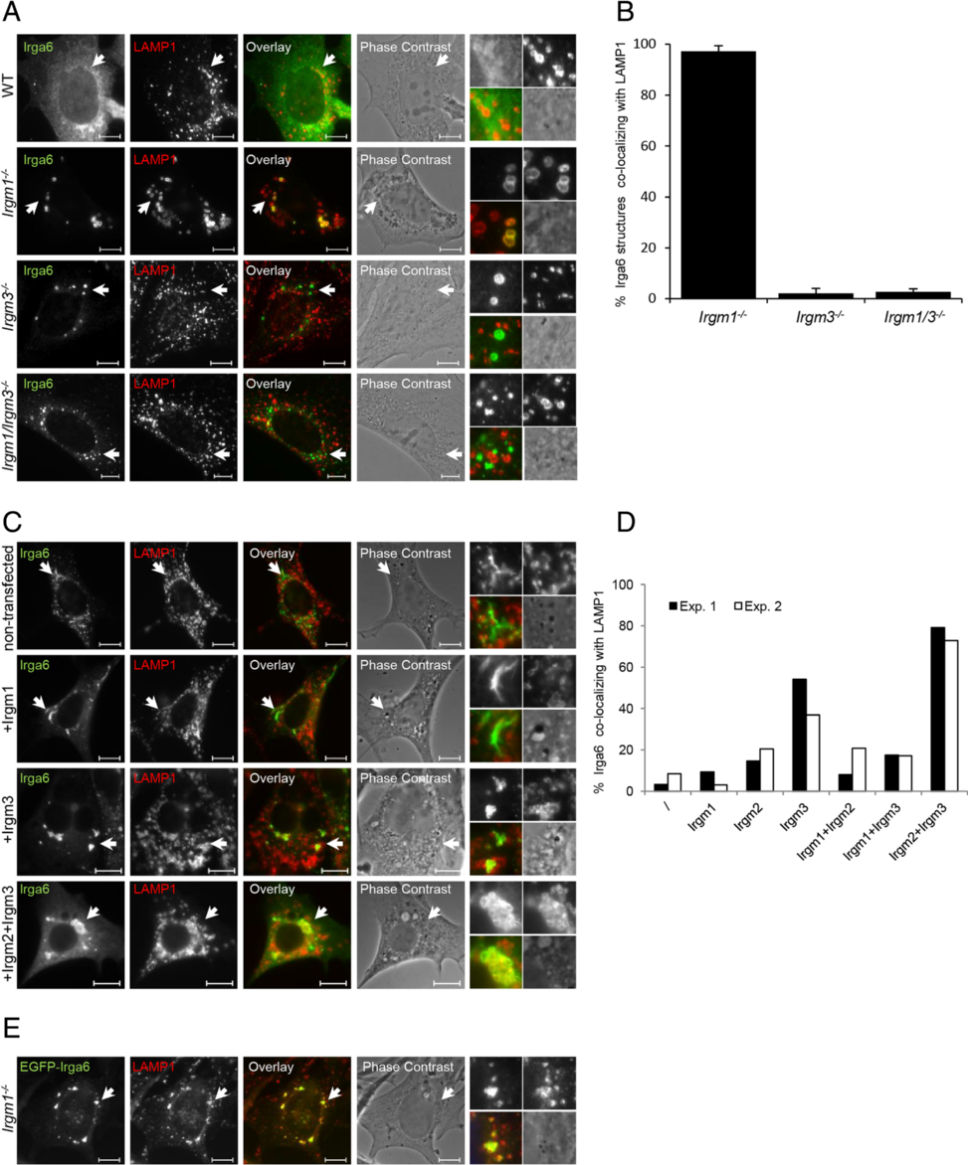
In the absence of Irgm1, Irga6 co-localizes with lysosomes. **a** Wild type (WT), *Irgm1* ^−/−^, *Irgm3* ^−/−^, and *Irgm1/Irgm3* ^−/−^ mouse embryonic fibroblasts (MEFs) were induced with 200 U/mL IFN-γ for 24 hours. Cells were fixed and stained with anti-Irga6 antiserum (165/3) and anti-LAMP1 antibody. Representative microscopic images of Irga6 and lysosome co-localization are shown. Arrows point at the Irga6 structures magnified at the end of each panel in the following array: upper left: Irga6, upper right: LAMP1, lower left: merge, lower right: phase contrast. Scale bars represent 10 μM. **b** Quantification of 2a, showing percentage of Irga6 aggregate-like structures co-localizing with LAMP1. Irga6 does not form aggregate-like structures in WT cells and therefore it was not quantified; 100 cells per sample were quantified and the means of three independent experiments ± standard deviation are shown. **c** Gene switch (gs) 3T3 cells stably transfected with inducible Irga6 were stimulated with mifepristone and simultaneously transiently transfected with pGW1H-Irgm1, pGW1H-Irgm2, and pGW1H-Irgm3 either alone or in combination and incubated for 24 hours. Samples were fixed and stained as in 1a. Representative images of Irga6 and lysosome co-localization are shown. Images of the cells transfected with additional combinations of GMS proteins are also included (Additional file 2: Figure S2). Arrows point at Irga6 structures magnified at the end of each panel in the following array: upper left: Irga6, upper right: LAMP1, lower left: overlay, lower right: phase contrast. Scale bars represent 10 μm. **d** Quantification of 2c and S2, showing percent of Irga6 structures co-localizing with LAMP1; 50 cells per sample were quantified and counts of two independent experiments are shown. **e** *Irgm1* ^−/−^ MEFs were induced with 200 U/mL IFN-γ and simultaneously transiently transfected with pEGFP-Irga6-ctag. Upon 24 h cells were fixed and stained for LAMP1. Scale bars represent 10 μM

To test this idea, immortalized WT mouse embryonic fibroblasts (MEFs), *Irgm1*^−/−^ MEFs, *Irgm3*^−/−^ MEFs, and *Irgm1/Irgm3*^−/−^ MEFs, all on a C57BL/6 background, were induced with IFN-γ for 24 hours and stained for Irga6 and for the late endosome/lysosome marker lysosomal-associated membrane protein 1 (LAMP1). In IFN-γ-induced WT MEFs, Irga6 retained the typical smooth non-aggregated pattern associated with the inactive state [17]. Irga6 formed aggregates in all GMS mutant cells, but clear ring-like forms co-localized with LAMP1 only in *Irgm1*^−/−^ cells (Fig. 2a, b). However, in *Irgm1/Irgm3*^*−/−*^ cells, in which both lysosomes and ER should lack GMS proteins, Irga6 aggregates do not co-localize with either of these cytoplasmic structures but co-localize with lipid droplets, confirming a recent report [21] (Fig. 2a). The Irga6 accumulated at lysosomes in Irgm1-deficient cells was shown to be in the activated state by intense staining with the monoclonal antibody 10D7 (Additional file 1: Figure S1), shown previously to bind preferentially to the active state of Irga6 [33].

To confirm that Irga6 localization to lysosomes is dependent specifically on Irgm1 deficiency, we have used another approach previously employed to demonstrate the regulatory function of GMS proteins [16]. Mouse 3T3 fibroblasts carrying stable transfected inducible constructs expressing Irga6 (Gene Switch (gs) 3T3-Irga6) were induced with mifepristone (MIF) and simultaneously transiently transfected with combinations of vectors expressing Irgm1, Irgm2, or Irgm3 (Fig. 2c, Additional file 2: Figure S2). These cells were not induced with IFN-γ and thus did not express endogenous IRG proteins, but only the induced Irga6 and the transfected GMS proteins. To distinguish transiently transfected from non-transfected cells, cytosolic pmCherry was co-transfected with the GMS constructs. Co-localization of Irga6 and LAMP1 was analyzed in mCherry-positive cells. In cells transfected with both Irgm2 and Irgm3, analogous to the GMS situation in *Irgm1*^−/−^ cells, more than 70 % of Irga6 accumulations co-localized with LAMP1 structures (Fig. 2d). In cells transfected only with Irgm3, more than 35 % of Irga6 accumulations were also co-localized with LAMP1, while in cells transfected only with Irgm1 or in non-transfected MIF-induced cells, less than 10 % Irga6 accumulations co-localized with LAMP1. As previously shown [16], when Irgm1, Irgm2, and Irgm3 were all co-transfected into MIF-induced cells, Irga6 showed the smooth non-aggregated appearance of the inactive state (Additional file 2: Figure S2).

To test whether transiently transfected EGFP-tagged Irga6 localizes to LAMP1 structures in the same manner as endogenous Irga6, *Irgm1*^−/−^ MEFs were induced with IFN-γ and simultaneously transfected with the pEGFP-Irga6 construct. EGFP-Irga6 indeed co-localized with lysosomes like endogenous Irga6 (Fig. 2e), indicating that EGFP-Irga6 can also be used for co-localization studies.

Taken together with the data in Fig. 2a, these findings show that Irga6 accumulates on LAMP1-positive organelles when Irgm1 is the only GMS protein absent from the cell.

### Other GKS proteins also localize to lysosomes in *Irgm1*^−/−^ cells

**Fig. 3:**
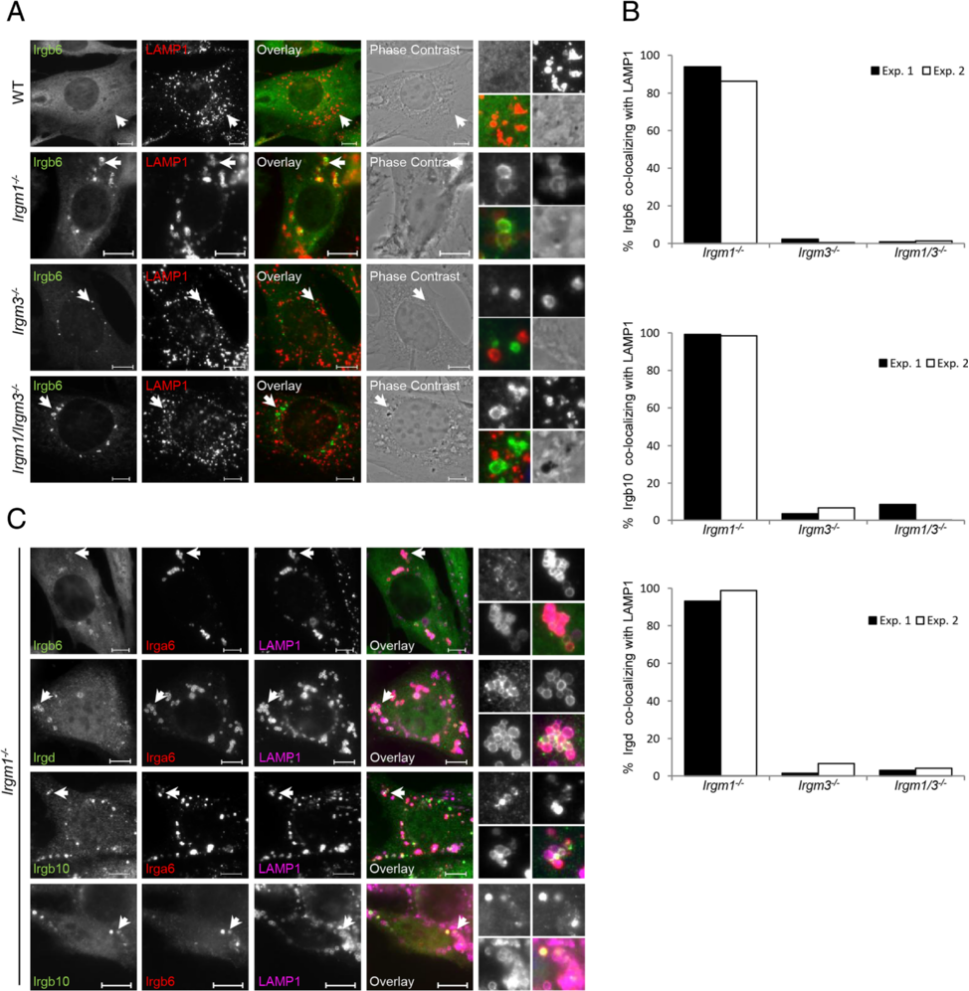
Other GKS proteins co-localize with lysosomes in *Irgm1* ^−/−^ cells. **a** Wild type (WT), *Irgm1* ^−/−^, *Irgm3* ^−/−^, and *Irgm1/Irgm3* ^−/−^ mouse embryonic fibroblasts (MEFs) were induced with 200 U/mL IFN-γ for 24 hours. Cells were fixed and stained with anti-Irgb6 antiserum (141/3) and anti-LAMP1 antibody. Representative microscopic images of Irgb6 and lysosome co-localization are shown. Arrows point at the Irgb6 structures magnified at the end of each panel in the following array: upper left: Irgb6, upper right: LAMP1, lower left: merge, lower right: phase contrast. Scale bars represent 10 μM. **b** Quantification of 3a, S3A and S3B, showing percent of Irgb6, Irgb10, and Irgd structures co-localizing with LAMP1; 50 cells per sample were quantified and results of two independent experiments are shown. **c** *Irgm1* ^−/−^ MEFs were induced with 200 U/mL IFN-γ for 24 hours, fixed and stained for Irga6 (10D7), Irgb6 (141/3) and LAMP1; Irgd, Irga6 (10D7) and LAMP1; Irga6 (10D7), Irgb10 and LAMP1; or Irgb6 (A20), Irgb10 and LAMP1. Representative microscopic images of GKS structures and lysosome co-localization are shown. Arrows point at the GKS structures which are magnified at the end of each panel in the following array: upper left: first GKS protein, upper right: second GKS protein, lower left: LAMP1, lower right: merge. Scale bar represents 10 μM

To investigate whether other GKS proteins also co-localize to LAMP1 in the absence of Irgm1, WT MEFs, *Irgm1*^−/−^ MEFs, *Irgm3*^−/−^ MEFs, and *Irgm1/Irgm3*^−/−^ MEFs were induced with IFN-γ and stained for the GKS proteins Irgb6, Irgb10, and Irgd and for LAMP1 (Fig. 3a, Additional file 3: Figure S3). More than 90 % of all GKS protein accumulations tested co-localized with LAMP1 in *Irgm1*^−/−^ cells and less than 5 % did so in *Irgm3*^−/−^ and *Irgm1/Irgm3*^−/−^ cells (Fig. 3b).

Loading of activated IRG proteins onto *T. gondii* vacuoles shows characteristics of cooperativity and hierarchy in the sense that several GKS proteins tend to load onto the same vacuoles and do so in a particular temporal order, in which Irga6 and Irgd can load only onto vacuoles already loaded with Irgb6 and/or Irgb10 [19]. We therefore tested the possibility of hierarchy and cooperativity of IRG loading onto lysosomes. *Irgm1*^−/−^ MEFs were induced with IFN-γ and stained for LAMP1 and for pairs of GKS proteins (Fig. 3c). Analysis of GKS protein combinations loaded at the lysosomes (Table 1) indicates a strict but distinct hierarchy in which Irga6 and Irgb10 can localize to the lysosome independently, while Irgb6 and Irgd can localize only to Irga6- and/or Irgb10-coated lysosomes. Irga6 and Irgb10 loading onto the lysosomes occur independent of each other. As observed for *T. gondii* vacuoles [19], not all LAMP1-positive organelles were coated with GKS protein accumulations. In these experiments, Irga6 was detected using 10D7 antibody, again confirming the activated state of Irga6 in the aggregates and accumulations.

**Table 1:** Cooperativity and hierarchy of IRG loading to lysosomes^a^

IRGs	Experiment	Total lysosomes	Lys. without IRGs	Lys. with 1^st^ IRG only	Lys. with 2^nd^ IRG only	Lys. with 1^st^ and 2^nd^ IRG	*P* value^b^	Independent or cooperative
Irga6 + Irgb6	1	1090	713	305	4	68	<0.0001	Cooperative
	2	1477	768	536	14	159	<0.0001	
Irga6 + Irgd	1	1692	880	503	18	291	<0.0001	Cooperative
	2	1232	702	299	8	223	<0.0001	
Irga6 + Irgb10	1	899	437	257	123	82	0.7995	Independent
	2	1337	570	459	184	124	0.5973	
Irgb10 + Irgb6	1	1060	866	144	2	48	<0.0001	Cooperative

^a^Lysosomes carrying or not carrying IRG proteins were identified microscopically by co-staining with antibodies against LAMP1 and different pairs of IRG proteins

^b^ *P* values were calculated in a χ^2^ analysis with 3 d.f. of the four categories of lysosome, based on expectation of random assortment of the two IRG proteins

### Irga6 co-localizes with endoplasmic reticulum in *Irgm3*^−/−^ cells

**Fig. 4:**
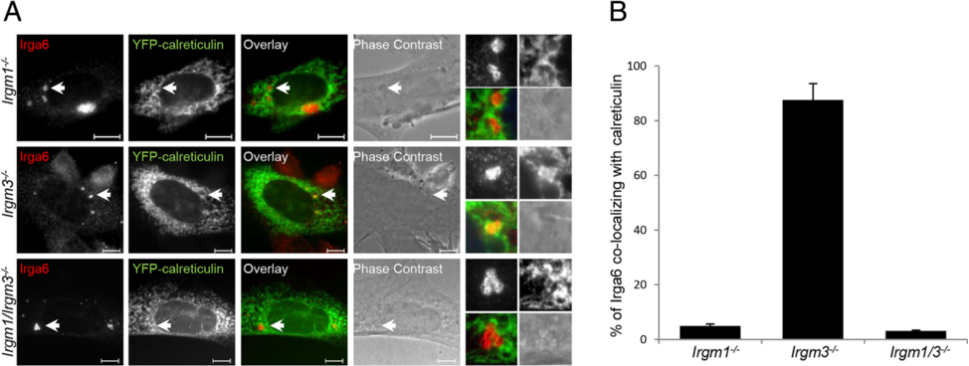
Irga6 co-localizes with the endoplasmic reticulum in *Irgm3* ^−/−^ cells. **a** *Irgm1* ^−/−^, *Irgm3* ^−/−^, and *Irgm1/Irgm3* ^−/−^ mouse embryonic fibroblasts (MEFs) were induced with 200 U/mL IFN-γ and transfected with pEYFP-calreticulin for 24 hours. Cells were fixed and stained with anti-Irga6 antibody (10D7). Representative microscopic images of Irga6 and calreticulin co-localization are shown. Arrows point at the Irga6 structures magnified at the end of each panel in the following array: upper left: Irga6, upper right: calreticulin, lower left: merge, lower right: phase contrast. Scale bars represent 10 μM. **b** Quantification of 4a, showing percent of Irga6 structures co-localizing with calreticulin; 50 cells per sample were quantified and the means of three independent experiments ± standard deviation are shown

Since Irgm3 is the only GMS protein identified as localizing to the ER [31], we asked whether, in the absence of Irgm3, Irga6 accumulates on the ER. WT, *Irgm1*^−/−^, *Irgm3*^−/−^_,_ and *Irgm1/Irgm3*^−/−^ MEFs were induced with IFN-γ and simultaneously transfected with an ER marker, pEYFP-calreticulin. The cells were again stained for activated Irga6 with 10D7 antibody (Fig. 4a). The Irga6 structures formed in GMS knock-out (KO) cells consist of activated, GTP-bound Irga6 [16, 33]. More than 85 % of active Irga6 accumulations co-localized with calreticulin in *Irgm3*^−/−^ cells (Fig. 4b). In contrast, fewer than 5 % of active Irga6 structures co-localized with calreticulin in *Irgm1*^−/−^ cells. These results strongly support the idea that the positioning of individual GMS proteins on specific endomembrane systems determines the inhibition of activation of GKS effector proteins on the same membranes.

### Irga6 co-localizes with lipid droplets in *Irgm1/Irgm3*^−/−^ cells

Most of the activated GKS proteins in *Irgm1*^−/−^ cells co-localize with the lysosomes and in *Irgm3*^*−/−*^ cells with the ER. The simple prediction is that both membrane systems will carry activated GKS proteins in the Irgm1/Irgm3 double KO. However, although these membranes should be GMS-free in *Irgm1/Irgm3*^−/−^ cells, neither is coated with GKS proteins (Figs. 2a and 4a). We therefore sought to confirm a recent report showing that in *Irgm1/Irgm3*^−/−^ cells activated GKS proteins co-localize with Bodipy-stained lipid droplets, to which both Irgm1 and Irgm3 both normally localize [21].

**Fig. 5:**
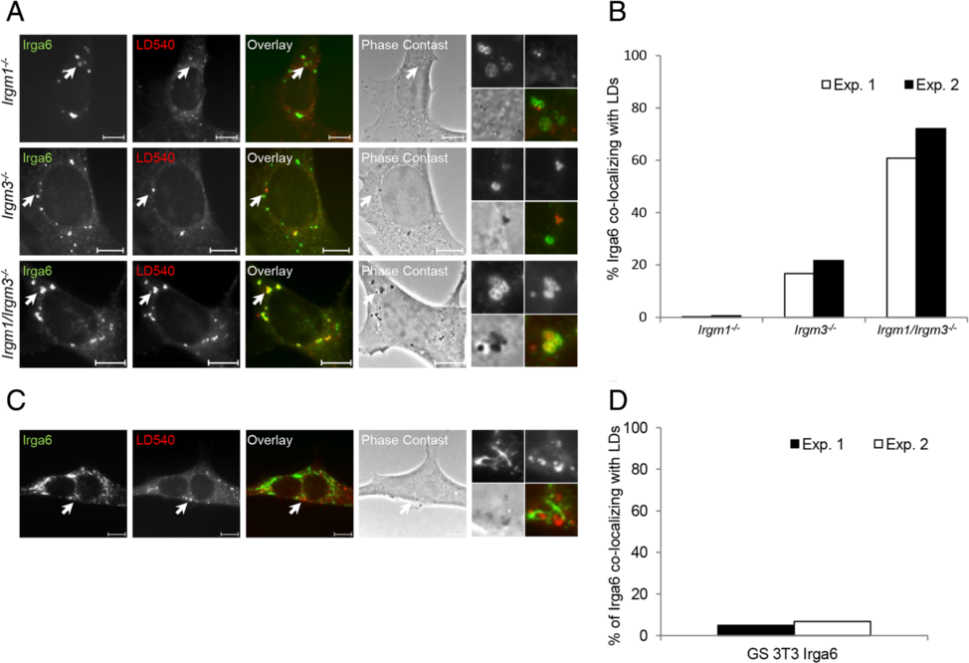
Irga6 co-localizes with lipid droplets in *Irgm1/Irgm3* ^−/−^ cells. **a** *Irgm1* ^−/−^, *Irgm3* ^−/−^, and *Irgm1/Irgm3* ^−/−^ mouse embryonic fibroblasts (MEFs) were induced with 200 U/mL IFN-γ for 24 hours. Cells were fixed and stained with anti-Irga6 antibody (10D7) and neutral lipid dye LD540. Representative microscopic images of Irga6 and lipid droplet co-localization are shown. Arrows point at the Irga6 structures magnified at the end of each panel in the following array: upper left: Irga6, upper right: LD540, lower left: phase contrast, lower right: merge. Scale bars represent 10 μM. **b** Quantification of 5a, showing percent of Irga6 structures co-localizing with LD540 (liquid droplets); 50 cells per sample were quantified and results of two independent experiments are shown. **c** gs3T3 cells stably transfected with inducible *Irga6* were stimulated with mifepristone for 24 hours. Samples were fixed and stained as in 5a. Representative microscopic images of Irga6 and LD540 co-localization are shown. Arrows point at the Irga6 structures magnified at the end of each panel in the following array: upper left: Irga6, upper right: LD540, lower left: phase contrast, lower right: merge. **d** Quantification of 5c, showing percent of Irga6 structures co-localizing with lipid droplets; 50 cells per sample were quantified and results of two independent experiments are shown

IFN-γ-induced *Irgm1*^−/−^, *Irgm3*^−/−^, and *Irgm1/Irgm3*^−/−^ MEFs were stained with anti-Irga6 antibody and with the neutral lipid dye LD540 (Fig. 5a). In accordance with the previous report [21], about 70 % of Irga6 structures co-localized with lipid droplets in *Irgm1/Irgm3*^−/−^ MEFs, less than 20 % did so in *Irgm3*^−/−^ cells, and almost none did so in *Irgm1*^−/−^ cells (Fig. 5b).

It has not been reported where activated Irga6 localizes in MIF-induced gs3T3 Irga6 cells in the absence of all GMS proteins. We tested the possibility that activated Irga6 accumulates on lipid droplets in the current conditions. MIF-induced gs3T3 Irga6 cells were stained with anti-Irga6 antibody and with LD540 dye (Fig. 5c). Less than 5 % of Irga6 aggregates were co-localized with the lipid droplets, indicating that this is not the primary GKS-target compartment in gs3T3 cells (Fig. 5d). We could also show that Irga6 does not co-localize with mitochondria in gs3T3 cells (Additional file 4: Figure S4). The compartment in which Irga6 activates in the absence of all GMS proteins remains unidentified.

### Irga6 does not co-localize with the Golgi in the absence of GMS proteins

The Golgi complex in IFN-γ-induced WT cells is normally coated with both Irgm1 and Irgm2. Because of the presence of Irgm2, the Golgi therefore should not be GMS-free in *Irgm1*^*−/−*^, *Irgm3*^*−/−*^, or *Irgm1/Irgm3*^*−/−*^ cells. To test whether GKS proteins accumulate at the Golgi in GMS KO cells, these cells were induced with IFN-γ and stained for the Golgi marker GM130 and for Irga6. As anticipated, in all GMS KO cells, less than 3 % of Irga6 co-localized with Golgi (Additional file 5: Figure S5). However, to analyze GKS co-localization with the Golgi in cells with other GMS protein combinations, gs3T3 cells were induced with MIF to express Irga6, transfected with combinations of different GMS proteins and stained for the Golgi marker GM130 and Irga6. In none of the samples, even in those where the Golgi should be GMS-free, was there co-localization of Irga6 with GM130 (Additional file 6: Figure S6). Thus, protection of Golgi membranes from ectopic activation of GKS proteins cannot apparently be accounted for by the presence of GMS proteins.

### *Irgm1*^−/−^ MEFs show autophagic flux impairment and defective lysosomal degradation

A variety of infections that increase IFN-γ levels in mice and induce IRG protein expression cause striking leukopenia and death in *Irgm1*^−/−^ mice but not in *Irgm1/Irgm3*^*−/−*^ mice [35]. Since GKS proteins re-localize to lysosomes in *Irgm1*^−/−^ MEFs but not in *Irgm1/Irgm3*^*−/−*^ MEFs, we investigated whether GKS protein activation on lysosomes can cause lysosomal dysfunction. This could contribute to the systemic *Irgm1*^−/−^ mouse phenotype.

**Fig. 6:**
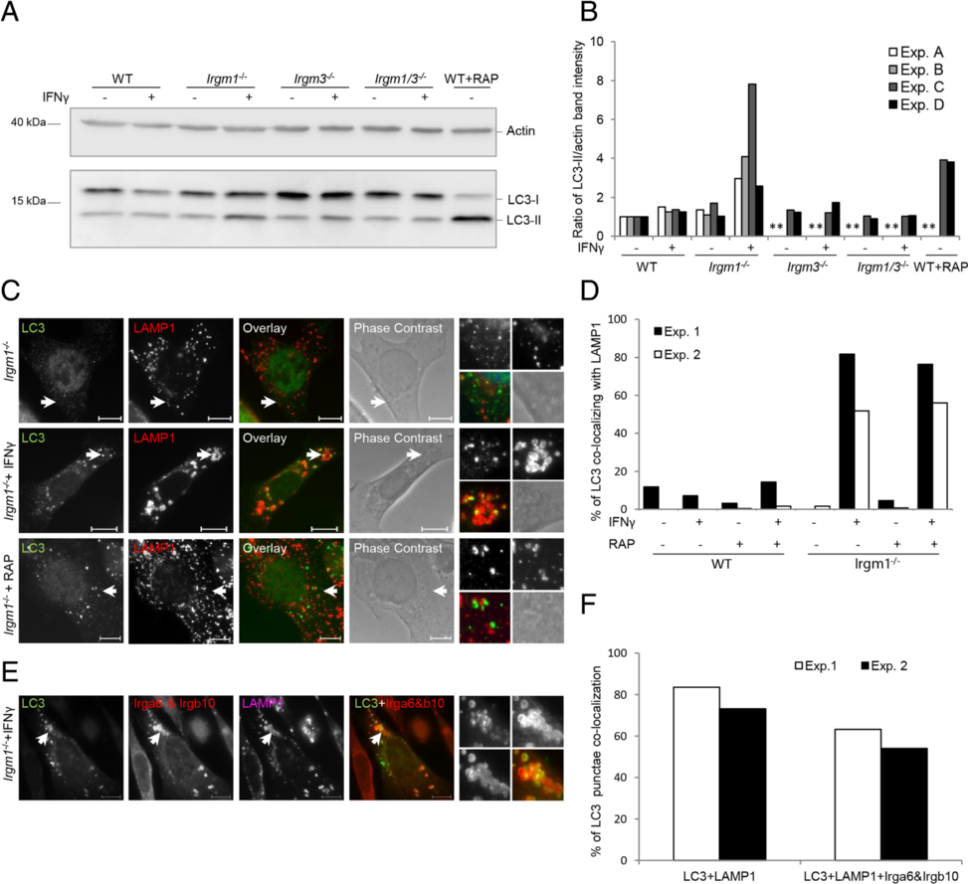
IFN-γ-induced *Irgm1* ^−/−^ mouse embryonic fibroblasts (MEFs) show autophagic flux impairment. **a** WT, *Irgm1* ^−/−^, *Irgm3* ^−/−^, and *Irgm1/Irgm3* ^−/−^ MEFs were induced with 200 U/mL IFN-γ for 24 hours, 40 μg/mL rapamycin (RAP) for 2 hours or left untreated. Samples were lysed and equal sample amounts were analyzed by SDS-PAGE/western blot. Western blots were probed with anti-LC3 and anti-actin antibody. **b** Quantification of 6a, representing ratios of LC3-II and actin band intensities for each sample. Results of four independent experiments are shown. Asterisks mark samples that were not included in the specific experiment. **c** Wild type (WT) and *Irgm1* ^−/−^ MEFs were induced with 200 U/mL IFN-γ for 24 hours, 40 μg/mL RAP for 2 hours, or left untreated. Cells were fixed and stained for LC3 and LAMP1. Representative microscopic images of LC3 and LAMP1 co-localization are shown. Arrows point at the LC3 structures magnified at the end of each panel in the following array: upper left: LC3, upper right: LAMP1, lower left: merge, lower right: phase contrast. Scale bars represent 10 μM. **d** Quantification of 6c and S7, showing percent of LC3 structures co-localizing with LAMP1; 50 cells per sample were quantified and the results of two independent experiments are shown. **e** WT and *Irgm1* ^−/−^ MEFs were induced with 200 U/mL IFN-γ for 24 hours or left untreated and transfected with EGFP-LC3. Cells were fixed and stained for LAMP1, Irga6 (165/3), and Irgb10. Irga6 and Irgb10 were detected with the same secondary antibody (Donkey anti-rabbit Alexa555), so they both appear in the same channel. Representative microscopic images of LC3, LAMP1, and Irga6/Irgb10 co-localization are shown. Arrows point at the LC3 structures shown in enlargement at the end of each panel in the following array: upper left: LC3, upper right: Irga6 and Irgb10, lower left: LAMP1, lower right: Merge of Irga6, Irgb10, and LC3. Scale bars represent 10 μM. **f** Quantification of 6e, showing percent of LC3 structures co-localizing with LAMP1 and percent of LC3 structures co-localizing with Irga6, Irgb10, and LAMP1; 50 cells per sample were quantified and the results of two independent experiments are shown

Autophagosomes are degraded by fusion with lysosomes, resulting in a flux of autophagic material through this compartment [51]. It has been previously reported that levels of p62 protein, which directs ubiquitinated substrates for autophagic degradation, are increased in IFN-γ-induced *Irgm1*^*−/−*^ cells as a result of autophagic flux impairment [48]. Moreover, an increased number of microtubule associated protein 1 light chain 3 (LC3) punctae has been shown in *Irgm1*^*−/−*^ HSCs, also indicating autophagic changes [47]. To better understand these autophagic flux alterations, processing of LC3, seen as LC3-I to LC3-II turnover, was monitored by western blot analysis (Fig. 6a). WT, *Irgm1*^−/−^, *Irgm3*^−/−^, and *Irgm1/Irgm3*^−/−^ MEFs were treated with IFN-γ, with the autophagy inducer rapamycin (RAP), or left untreated. The intensity of the LC3-II band was noticeably increased only in IFN-γ-treated *Irgm1*^−/−^ MEFs in comparison to the other samples (Fig. 6b), a result consistent with the behavior of p62 in an earlier study [48]. However, the intensity of LC3-I was not reduced in these cells. Increase in LC3-II could be an outcome of two scenarios: firstly, autophagy induction might be enhanced in IFN-γ-treated *Irgm1*^−/−^ cells; secondly, autophagic flux might be impaired in the lysosomes of these cells so that LC3-II cannot be further processed and degraded. The fact that the LC3-I level was not reduced in these cells supports the second scenario. We therefore investigated LC3 distribution in *Irgm1*^−/−^ cells in more detail. WT MEFs and *Irgm1*^−/−^ MEFs were treated with IFN-γ and/or RAP or left untreated, and further stained with LC3 antibody (Additional file 7: Figure S7). As expected, LC3 formed a large number of puncta-like structures in RAP-treated cells and only few small punctae in non-treated cells. However, a large number of LC3 punctae could be observed in IFN-γ-induced *Irgm1*^−/−^ MEFs, even though they were not RAP induced, indicating an increased number of autophagosomes.

We next analyzed the co-localization of LC3 punctae with LAMP1-positive structures in these cells. In all WT MEFs and in untreated or RAP-treated *Irgm1*^−/−^ MEFs, very few LC3-positive structures co-localizing with lysosomes were observed, even when a large number of LC3 punctae was present in RAP-treated cells. However, in IFN-γ-induced *Irgm1*^−/−^ cells, independently of RAP treatment, 60–80 % of LC3 punctae co-localized with lysosomes (Fig. 6c, d; Additional file 8: Figure S8). This suggested that autophagosomes cannot be further processed and degraded after fusion with lysosomes in IFN-γ-induced *Irgm1*^−/−^ cells.

We then asked whether the lysosomes that are unable to process LC3 are the same lysosomes that are coated with activated GKS proteins. *Irgm1*^−/−^ MEFs were induced with IFN-γ, transfected with EGFP-LC3, and stained for LAMP1, Irga6, and Irgb10 (Fig. 6e). As in the previous experiment, a large percentage of LC3 co-localized with LAMP1 in IFN-γ-induced *Irgm1*^−/−^ cells and a large percentage of these LC3 punctae co-localizing with LAMP1 were also associated with GKS proteins Irga6 and Irgb10 (Fig. 6f). It therefore seems plausible that the LC3 processing impairment in *Irgm1*^−/−^ cells is caused by the accumulation of activated GKS proteins on the lysosomes. The autophagosomal compartments with impaired function in the Irgm1-deficient cells have clearly fused with lysosomes since they are LAMP1 positive. In this respect, our results differ significantly from Traver et al. [48], who were unable to detect LAMP1 staining associated with the Irga6 positive, Gbp2 positive aggregates, while our data show that activated Irga6 in the Irgm1-deficient cells is almost exclusively associated with LAMP1 positive structures.

**Fig. 7:**
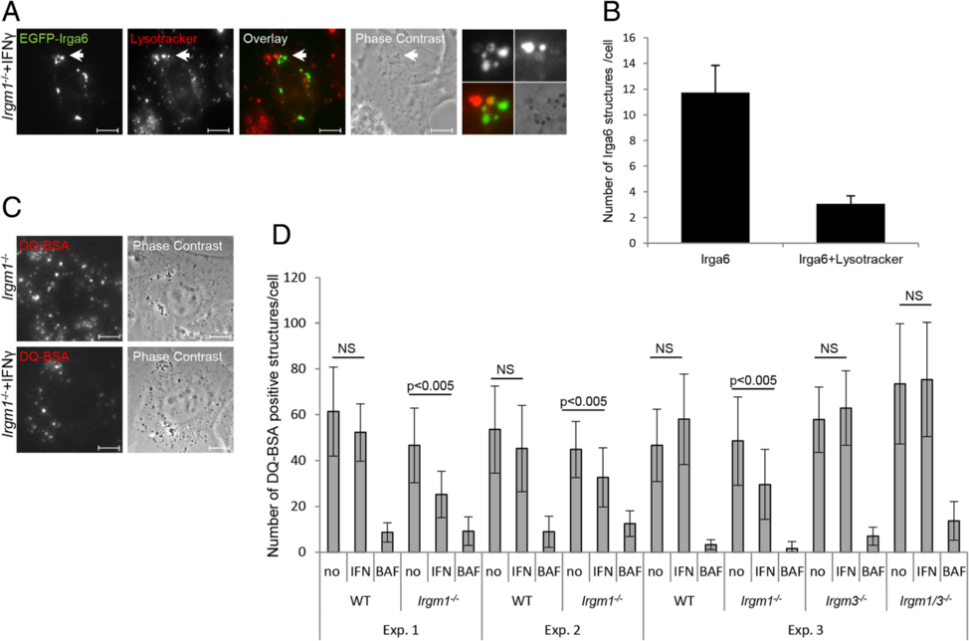
Lysosomal processing is impaired in IFN-γ-induced *Irgm1* ^*−/−*^ mouse embryonic fibroblasts (MEFs). **a** *Irgm1* ^−/−^ MEFs were transfected with EGFP-Irga6 and simultaneously induced with 200 U/mL IFN-γ. After 24 hours, non-fixed cells were incubated with 50 nM Lysotracker for 15 minutes and pictures of live cells were taken. Representative microscopic images of Irga6 and Lysotracker co-localization are shown. Arrows point at the Irga6 structures magnified at the end of each panel in the following array: upper left: Irga6, upper right: Lysotracker, lower left: merge, lower right: phase contrast. Scale bars represent 10 μM. **b** Quantification of 7a showing the number of Irga6 structures co-localizing with Lysotracker. 25 cells per sample were quantified and the means of three independent experiments ± standard deviation are shown. **c** Wild type (WT), *Irgm1* ^−/−^, *Irgm3* ^−/−^, and *Irgm1/Irgm3* ^−/−^ MEFs were induced with 200 U/mL IFN-γ for 27 hours, 200 nM μg/mL Bafilomycin A1 for 4 hours, or left untreated. After 24 hours, non-fixed cells were incubated with 10 μg/mL DQ-BSA, incubated for 3 hours, and images of live cells were taken. Representative microscopic images of DQ-BSA staining are shown. Scale bars represent 10 μM. **d** Quantification of Fig. 7c showing the average numbers of DQ-positive structures per cells; 20 cells per sample were evaluated and results of three independent experiments ± standard deviation are shown. Differences were tested for statistical significance using the Mann-Whitney test (NS *P* > 0.03)

Since lysosomes of IFN-γ-induced *Irgm1*^−/−^ cells were apparently not able to process autophagosomes, we asked whether the general processing activity of these lysosomes is impaired. We first analyzed the pH status of Irga6-coated lysosomes with the pH sensitive lysosomal dye Lysotracker on live, non-fixed cells. *Irgm1*^−/−^ MEFs were transfected with EGFP-Irga6 and simultaneously induced with IFN-γ (Fig. 7a). EGFP-Irga6 structures that co-localized with Lysotracker were quantified (Fig. 7b). Out of 11.7 Irga6 structures per cell, only 3.1 were Lysotracker-positive. We show above that EGFP-Irga6 structures co-localize completely with LAMP1 (Fig. 2e). Therefore, the data show that the pH of the majority of Irga6-coated lysosomes in IFN-γ-induced *Irgm1*^−/−^ cells is raised, resulting in an impairment of autophagosome processing.

We also analyzed lysosomal degradation of DQ-BSA [52]. In this substrate, a fluorescent dye is quenched by the protein carrier, but revealed when the substrate is processed in lysosomes. WT, *Irgm1*^−/−^, *Irgm3*^−/−^, and *Irgm1/Irgm3*^−/−^ MEFs were treated with IFN-γ, with the lysosomal acidification inhibitor Bafilomycin A1 (BAF), or left untreated. Samples were incubated with DQ-BSA. The numbers of DQ-positive punctae were evaluated blind on pictures of live cells (Fig. 7c). IFN-γ-induced *Irgm1*^*−/−*^ MEFs had 30–50 % fewer punctae than non-induced *Irgm1*^*−/−*^ MEFs (Fig. 7d). This difference was not observed in WT, *Irgm3*^−/−^, or *Irgm1/Irgm3*^−/−^ MEFs. As expected, the number of DQ-punctae in BAF-treated cells was reduced by more than 80 %. Thus, it seems that GKS-coated lysosomes are not only impaired in autophagosomal processing, but also in processing of exogenous substrates.

### GKS proteins probably do not permeabilize lysosomal membranes in *Irgm1*^−/−^ cells

After observing that GKS proteins localize to LAMP1 structures in *Irgm1*^*−/−*^ cells, we considered the possibility that these GKS proteins might disrupt the lysosomal membrane in in the same way that they mediate disruption of the *T. gondii* PVM. Hence, the outcome of GKS accumulation to the lysosomes could be lysosomal membrane permeabilization (LMP). This would cause not only breakdown of the pH gradient but also lysosomal enzyme leakage and presumably death of the cell [53].

**Fig. 8:**
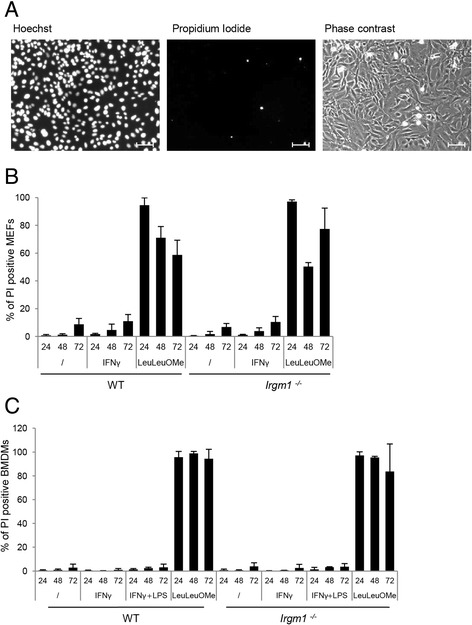
*Irgm1* ^−/−^ mouse embryonic fibroblasts (MEFs) and bone marrow derived macrophages (BMDMs) do not die upon IFN-γ induction. **a** Wild type (WT) and *Irgm1* ^−/−^ MEFs were induced with 200 U/mL IFN-γ or treated with 10 mM LeuLeuOMe for 24, 48, and 72 hours, or left untreated. Cells were stained with nuclear dyes Hoechst (all cells) and propidium iodide (PI; dead cells only). Representative microscopic images of Hoechst- and PI-stained WT cells are shown. Scale bar represents 100 μM. **b** Quantification of 8a showing the percentage of PI-positive MEFs. Dye stained nuclei were quantified with the Volocity software; 10 pictures (5000–10000 cells) per sample were quantified and the means of three independent experiments ± standard deviation are shown. **c** WT and *Irgm1* ^−/−^ BMDM cells were induced with 200 U/mL IFN-γ, 500 ng/mL lipopolysaccharide, 5 mM LeuLeuOMe for 24, 48, and 72 hours, or left untreated. The experiment was performed as in 8a, b; 10 pictures (5000–10000 cells) per sample were quantified and the means of three independent experiments ± standard deviation are shown

To analyze the possibility of necrotic cell death, WT and *Irgm1*^−/−^ MEFs were induced with IFN-γ or left untreated. Treatment with the LMP inducer LeuLeuOMe [54, 55] was used as a positive cell death control. After 24, 48, or 72 hours, live cells were stained with cell-permeant DNA binding Hoechst dye, which stains the nuclei of live and dead nuclei, and cell-impermeant propidium iodide (PI), which stains only the nuclei of dead cells (Fig. 8a). No difference in cell death between WT and *Irgm1*^−/−^ MEFs was observed (Fig. 8b). We repeated this experiment in WT bone marrow derived macrophages (BMDM) and *Irgm1*^−/−^ BMDMs, which were induced with IFN-γ, lipopolysaccharide, LeuLeuOMe, or left untreated (Fig. 8c). As previously reported in a similar analysis [6], no difference in cell death between the two cell populations was observed.

Taken together, even though it is a characteristic of activated *Irgm1*^−/−^ lymphocytes [35], necrotic cell death was not observed in IFN-γ-induced *Irgm1*^−/−^ MEFs and BMDMs. Moreover, lack of cell death in these samples largely excludes the possibility of lysosomal membrane permeabilization.

To further consider the possibility that GKS proteins cause lysosomal permeabilization in *Irgm1*^*−/−*^ cells without causing massive necrosis, we analyzed the release of the lysosomal enzyme cathepsin B into the cytosol (Additional file 9: Figure S9) compared with the release induced by a just sub-lethal concentration of the pronecrotic peptide LeuLeuOMe.

WT MEFs and *Irgm1*^*−/−*^ MEFs were left un-treated, induced with IFN-γ, or treated with 3 mM LeuLeuOMe. Cells were fixed and stained for cathepsin B (Additional file 9: Figure S9C) and the small, sharply-defined cathepsin B punctae indicating intact lysosomes counted (Additional file 9: Figure S9D). Both, LeuLeuOMe-treated WT and LeuLeuOMe-treated *Irgm1*^−/−^ cells had fewer cathepsin B punctae than non-treated cells and also contained localized, larger diffuse stained regions presumably indicating local lysosomal damage and cathepsin B release. However, the number of sharply-defined cathepsin B punctae in IFN-γ-induced *Irgm1*^−/−^ MEFs was very similar to untreated *Irgm1*^−/−^ MEFs as well as treated and untreated WT MEFs, and the characteristic diffusely stained regions found in the LeuLeuOMe-treated cells were not seen. Taken together, these data indicate that GKS proteins, which are localized to lysosomes, do not mediate significant lysosomal membrane permeabilization in IFN-γ-induced *Irgm1*^*−/−*^ fibroblasts and macrophages.

## Discussion

The argument that Irgm1 is an important pathogen resistance molecule in mice has largely relied on the dramatic loss of resistance to numerous pathogens resulting from elimination of *Irgm1* by gene targeting. This interpretation has persisted despite the clear demonstration that Irgm1 deficiency has large systemic effects on the lymphomyeloid system. Lymphomyeloid collapse is induced by infection in Irgm1-deficient animals [34] caused, at least in part, by constitutive exhaustion of HSCs in these animals [45]. Loss of general pathogen resistance is thus most plausibly accounted for by the profound damage to the lymphomyeloid system in Irgm1 deficiency. The critical question is thus not how Irgm1 functions as a pathogen resistance molecule, but rather why its absence causes this dramatic phenotype. The issue was further complicated when Taylor and colleagues showed that loss of a second regulatory member of the IRG system, Irgm3, almost completely rescued the Irgm1 deficiency phenotype, both in terms of lymphomyeloid collapse and pathogen resistance [6]. Absence of Irgm1, per se, could therefore not be responsible for the problem. However, the Irgm1/Irgm3 double-deficient animals were not completely normal: despite a normal lymphomyeloid system, they remained fully susceptible to *Toxoplasma gondii* [6] and their cells cannot inhibit *E. cuniculi* replication after IFN-γ treatment [14]. Indeed, Irgm1 and Irgm3 are functional regulators of the IRG resistance system [16] and since their absence leaves animals vulnerable to those few pathogens that are controlled by the IRG resistance system, they may legitimately be described as resistance factors. However, the catastrophic phenotype of the Irgm1-alone-deficient mouse is clearly something different, and it was the motive of this study to point to the causal chain leading to this dramatic outcome.

It has been proposed that effector proteins of the IRG system (GKS proteins) target pathogen-containing vacuoles in IFN-γ-induced cells through the absence on the vacuolar membranes of the GMS regulatory proteins of the IRG system [14–16, 20–22]. In this model, GKS proteins would indeed tend to activate on endomembrane systems, but would normally be inhibited by GMS proteins localized to these compartments. This would prevent potential damage caused by off-target activation of the GKS proteins on self-membranes and leave the PVM of the pathogen, as the only GMS-free membrane in the cell, thus exposed as a target for the effector GKS proteins. Since different GMS proteins localize to different endocellular membranes, absence of a specific GMS protein would leave a specific endocellular membrane unprotected and vulnerable to off-target activation of GKS proteins.

**Fig. 9:**
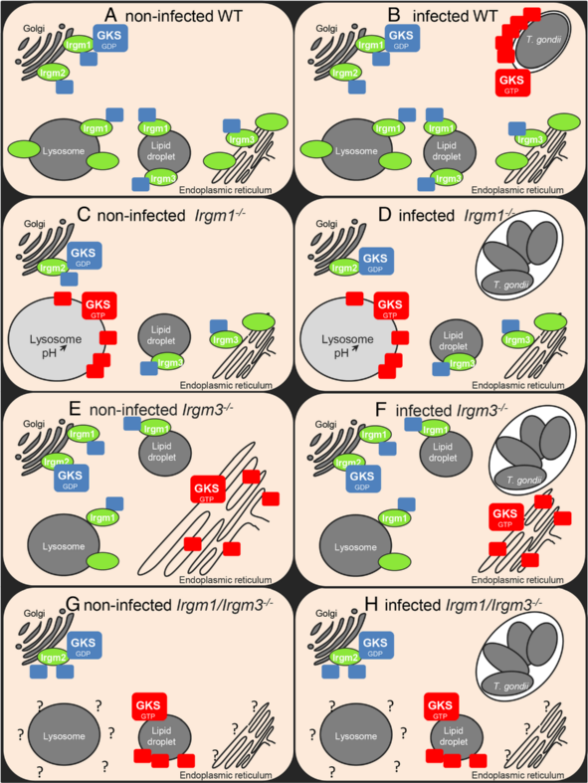
Model for the cytopathic effects of dysregulated GKS proteins. **a** Regulatory immunity-related GTPase (IRG) GMS proteins localize to distinct cellular endomembranes including the endoplasmic reticulum (ER), Golgi, lipid droplets, and lysosomes and maintain effector IRG GKS proteins, which diffuse transiently onto these compartments, in an inactive GDP-bound state. **b** When *T. gondii* enters the host cell, GKS proteins diffuse onto this new membrane-bound compartment and activate at the parasitophorous vacuole membranes (PVM) because of the absence of GMS proteins. Activated GKS proteins form GTP-dependent oligomers and disrupt the membrane. **c** In Irgm1-deficient cells, lysosomal membranes lack any GMS proteins on the lysosomal membrane, allowing GKS proteins to activate and accumulate forming GTP-bound oligomers. Acidification of these lysosomes is impaired and with it lysosomal processing is not functional. These lysosomes cannot properly process autophagosomes after lysosome/autophagosome fusion. Hence, autophagic flux of IFN-γ-induced *Irgm1* ^−/−^ cells is impaired. **d** In *T. gondii*-infected *Irgm1* ^*−/−*^ cells, GKS proteins are accumulated at the lysosomes and therefore cannot properly accumulate and activate at the PVM. Disruption of the PVM does not happen and *T. gondii* can further proliferate. **e** In Irgm3-deficient cells it is the ER cisternae that are not protected by GMS proteins. Activated GKS proteins accumulate in local aggregates on the ER, possibly causing local ER deformation, but without such severe consequences for the cell as lysosomal impairment in *Irgm1* ^−/−^ cells. **f** In *T. gondii*-infected *Irgm3* ^*−/−*^ cells, GKS proteins are accumulated at the ER and therefore cannot properly accumulate and activate at the PVM. Disruption of the PVM does not happen and *T. gondii* can proliferate. **g** In *Irgm1/Irgm3* ^*−/−*^ cells, lysosomes, ER, and lipid droplets are GMS-free. For unknown reasons, activated GMS proteins accumulate only at the lipid droplets and not at the other GMS-free compartments. **h** Upon *T. gondii* infection of *Irgm1/Irgm3* ^*−/−*^ cells, GKS proteins activated at the lipid droplets cannot properly load to the PVM and inhibit *T. gondii* proliferation

In this study, we have investigated the possibility proposed in our earlier publications [16, 20] that the contrasting phenotypes of Irgm1, Irgm3, and the Irgm1/Irgm3 double deficient mice might be caused by off-target activation of the GKS proteins at different cellular endomembranes. We have shown that the localization of activated GKS proteins to specific compartments is indeed dependent on the absence of specific GMS proteins (Fig. 9). In IFN-γ induces cells in the absence of Irgm1, activated GKS proteins accumulate at the lysosomes (Fig. 2a, b), to which Irgm1 normally localizes, while in the absence of Irgm3, activated GKS proteins accumulate at the ER (Fig. 4), to which Irgm3 normally localizes. In *Irgm1/Irgm3*^*−/−*^ cells, in which lysosomes, ER, and lipid droplets are all GMS-free, GKS proteins activate exclusively at lipid droplets. Thus, the activation of GKS proteins on specific endomembrane systems is determined by the absence of specific GMS proteins (Fig. 5) [21]. However, the targeting specificity rules are evidently complex. Firstly, when single membrane systems, e.g., lysosomes alone or ER alone, are GMS-free, GKS activation occurs only on the GMS-free membranes. However, when multiple membrane systems are GMS-free, GKS activation occurs selectively on one set of GMS-free membranes and not on another. Thus, GKS proteins may show different preferences for different endocellular membranes when given a choice, perhaps on the basis of their lipid composition. To be clear, in the case of *Irgm1/Irgm3*^*−/−*^, we do not argue that Irgm3 normally interacts directly with Irgm1, or is in any sense a regulator of the localization of Irgm1, only that the additional absence of Irgm3 releases further compartments for access by GKS proteins. Secondly, in gs3T3 cells expressing Irga6 under an inducible promoter, but not transfected with GMS proteins, all endocellular membranes must be essentially GMS-free (Fig. 2c, d). If GKS proteins load randomly onto any GMS-free membrane, activated Irga6 should be found on lysosomes, ER, Golgi, and lipid droplets, but this is not the case. Instead, activated Irga6 localizes to a still unidentified punctate compartment which may, indeed, consist of freely cytosolic aggregates (Figs. 2d, 5d, and Additional file 6: Figure S6). Thirdly, when these gs3T3 Irga6 cells are transfected with Irgm3 only, about 35 % of Irga6 aggregates accumulate at the lysosomes. The Golgi complex, which is normally coated with Irgm1 and Irgm2, is also GMS-free in these cells. Irga6 would therefore also be expected to accumulate at Golgi membranes, but this does not happen. Indeed, the Golgi complex seems to be immune to GKS activation independently of GMS loading. Finally, none of the endocellular GMS proteins localizes to the plasma membrane, but this structure is never coated with GKS proteins, even in WT cells. The basis for this protection is not known [14]. However, whatever the critical properties of the plasma membrane are, they must change rapidly during pathogen entry because the new pathogen-containing vacuolar membrane immediately becomes vulnerable to attack by GKS proteins [19]. In summary, the activation of GKS proteins occurs only on GMS-free membranes, but not necessarily on all. Furthermore, although the location of activated GKS proteins depends upon which membranes are GMS-free, the rules of selectivity between potential target membranes remain unclear.

The consequences of GKS accumulation at different endocellular membranes are also not completely understood. Alteration of autophagic flux in the absence of Irgm1 in activated cells has already been proposed in different models [35, 47, 48]. Our novel claim is that this is due to the selective accumulation of activated GKS proteins on lysosomes due to the absence of Irgm1. Here, we show that the pH of GKS-coated lysosomes in cultivated Irgm1-deficient fibroblasts is increased and that these lysosomes cannot process autophagosomes, causing reduction in autophagic flux (Figs. 6 and 7). Lysosomes free of GKS accumulations in the same cells do not show an acidification defect. The lysosomes of IFN-γ-induced Irgm1-deficient cells also cannot process an exogenous substrate efficiently (Fig. 7).

Lymphocytes, which need to divide rapidly upon infection, are particularly challenged by autophagic flux arrest and lysosomal function failure [49, 50], and this may contribute to the fatal IFN-γ-induced lymphopenia in *Irgm1*^*−/−*^ mice. HSCs, which express IRG proteins in the absence of infection [56, 57], may be similarly vulnerable to this lesion in the Irgm1-deficient mouse [45, 47]. An increased number of microtubule associated protein 1 light chain 3 (LC3) punctae has been shown in *Irgm1*^*−/−*^ HSCs, indicating an autophagic anomaly [47] and fully consistent with evidence for constitutive expression of IRG proteins in this compartment. The consequences of GKS accumulation at the ER in *Irgm3*^*−/−*^ cells are not clear. It has been suggested that Irgm3 deficiency is associated with enlargement and swelling of this compartment [16, 58], but these mice do not become lymphopenic after infections that kill *Irgm1*^*−/−*^ mice. We must conclude that these ER anomalies do not affect the function of the lymphomyeloid system to the same extent as they damage the lysosomal system. It has been proposed that GKS proteins at the lipid droplets in *Irgm1/Irgm3*^*−/−*^ cells help the degradation of this compartment via autophagy [21]. These results require further investigation, but it is clear that GKS proteins in these cells are not at the lysosomes and that lysosomal and autophagic functions are not impaired. According to our model, this accounts for the fact that *Irgm1/Irgm3*^*−/−*^ mice do not show lymphopenia and do not die from the multitude of infections that kill *Irgm1*^*−/−*^ mice.

As we show, several members of the GKS subfamily load cooperatively on to lysosomes in Irgm1-deficient cells, just as several GKS members load on to *T. gondii*, *C. trachomatis*, and *E. cuniculi* vacuoles. It is curious that the priority rules for loading of different GKS proteins differ between *T. gondii* vacuoles and *Irgm1*^*−/−*^-deficient lysosomes. In no case is it known whether the consequent damage is due to some form of coordinated attack, whether the injury is additive, or whether certain specific GKS members are primarily responsible. In the present case, we consider it unlikely that the damage to the lysosomal pH gradient in fibroblasts and macrophages is due to frank disruption of the lysosomal membrane. The effect is not lethal while disruption of the lysosomal membrane would normally initiate necrotic death. In addition, there is no quantitative loss of cathepsin-containing organelles or evidence for release of cathepsins into the cytosol (Fig. 8). Recently, several studies have reported different aspects of the loading of guanylate binding proteins onto *T. gondii* PVMs, *C. trachomatis* inclusions, and IRG aggregate-like structures [21, 48, 59–62]. Guanylate binding proteins appear to load on to a subset of vacuoles that carry IRG proteins. A role in attracting the autophagic apparatus via ubiquitination has been proposed.

Whatever the basis for the damage to the pH gradient, the consequences of GKS protein off-target activation on endogenous organelles can evidently be severe and may indeed represent a risk and, therefore, a cost for possession of the IRG system. The spectrum of pathogens that are restricted by IRG proteins appears to be remarkably narrow [14]. Perhaps the loss of the IRG system in higher primates and certain other animal groups [2, 63, 64] is the response to a balance between the cost of carrying an immune resistance mechanism that can become dangerous and difficult to control, against the scale of threat posed by the limited pathogen classes that the mechanism defends against. Indeed, humans have what appears to be efficient resistance against most *T. gondii* strains despite their loss of the IRG system [2]. How humans distinguish between PVMs and endomembranes remains unknown.

## Conclusions

To summarize, the three members of the GMS subfamily of IRG resistance proteins in mice act as negative regulators, essentially guanine dissociation inhibitors, of the GKS effector subfamily. Their role is to prevent the GKS proteins from activating on endogenous organelles, while enabling them to accumulate on parasite-containing vacuoles, which are GMS deficient. Each of the three GMS proteins localizes to a different specific subset of intracellular organelles in the interferon-induced cell. The presence of GMS proteins thus enables GKS proteins to distinguish organellar “self” cellular membranes from the membranes of the pathogen vacuoles. The high susceptibility of *Irgm1*^−/−^ mice to multiple infections is caused by off-target GKS protein activation on lysosomal membranes, collapse of lysosomal pH gradient, and consequent failure of autophagosomal processing, a lesion to which members of the hematolymphoid cell lineage are particularly susceptible. Our experiments use the history of these phenomena to show a path of cause and effect between loss of Irgm1 and susceptibility to infection that is far more complex than the original, and still much cited view, that disease susceptibility of Irgm1-deficient animals shows that Irgm1 is an important general disease resistance factor.

## Methods

### Ethical statement

All animal experiments were conducted under the regulations and protocols for animal experimentation according to the German “Tierschutzgesetz” (Animal Experimentation Law). The local government authorities, Landesamt fuer Natur- und Umweltschutz Nordrhein Westfalen, and its ethics committee approved the work (LANUV Permit No. 84–02.05.40.14.004).

### Cell culture

Immortalized WT C57BL/6 MEFs, *Irgm1*^−/−^ MEFs, *Irgm3*^−/−^ MEFs, and *Irgm1/Irgm3*^−/−^ MEFs were prepared as previously described [14]. Cells were cultured in DMEM, high glucose (Life Technologies, Carlsbad, CA) supplemented with 10 % FCS (Biochrom, Berlin, Germany), 2 mM glutamine, 1 mM sodium pyruvate, 1× MEM non-essential amino acids, 100 U/mL penicillin, and 100 mg/mL Streptomycin (all Life Technologies).

gs3T3 Irga6 cells were prepared as previously described [16] and cultured in the above described medium with addition of 200 μg/mL Zeocin (Invivogen, San Diego, CA) and 50 μg/mL Hygromycin (Invivogen). Irga6 expression was induced with 1 nM mifepristone (Life Technologies).

Primary BMDMs were isolated from tibia and femurs of 4- to 5-week-old C57BL/6 mice and *Irgm1*^−/−^ mice as previously described [46], and cultured in RPMI 1640 (Life Technologies) supplemented with 20 % FCS, 30 % L 929 cell conditioned medium, 2 mM glutamine, 1 mM sodium pyruvate, 1× MEM non-essential amino acids, 100 U/mL penicillin, and 100 mg/mL streptomycin (all Life Technologies).

### Expression constructs and transfection

The following expression constructs were used: pGW1H-Irgm1 [19], pGW1H-Irgm2 [19], pGW1H-Irgm3 [19], pmCherry-N3 [7], pEGFP-Irga6-ctag1 [7], and N-tagged pEYFP-calreticulin (kindly provided by Dr. Astrid Schauss, CECAD Imaging Facility), pEGFP-LC3 [8].

Transient transfection was conducted with 1 μg DNA per 300,000 cells seeded using 3 μL of X-tremeGENE 9 transfection reagent (Roche, Mannheim, Germany) according to the manufacturer’s instructions.

### Immunological reagents and dyes

The following immunoreagents were used: rabbit anti-Irga6 antiserum 165/3 [17], mouse anti-Irga6 monoclonal antibodies (mAb) 10E7 and 10D7 [33], rabbit anti-Irgb6 antiserum 141/3 [65], antibody A20 (Santa Cruz Biotechnology, TX, USA), rabbit anti-Irgd antiserum 81/3 [65], anti-Irgb10 antiserum 940/6 [19], rat anti-LAMP1 monoclonal antibody 1D4B (Abcam, Cambridge, United Kingdom), anti-GM130 antibody (BD Biosciences, NJ, USA), rabbit anti-LC3B antiserum L7543 (Sigma-Aldrich, St. Louis, MO, USA), rabbit anti-Tom20 antiserum (Santa Cruz Biotechnology), mouse anti-β-Actin monoclonal antibody (Sigma-Aldrich), and mouse anti-cathepsin B antibody (R&D systems, MN, USA). The following secondary antibodies were used: Alexa Fluor 488/555/647-labeled donkey anti-mouse/donkey anti-rabbit/donkey anti-rat antibody (all Life Technologies), HRP-conjugated goat anti-mouse antibody and HRP-conjugated donkey anti-rabbit antibody (both Sigma-Aldrich). Nuclear staining was performed with 0.5 mg/mL DAPI (Life Technologies), 1 μg/mL PI (Sigma-Aldrich), or 1 μg/mL Hoechst 33342 (Sigma-Aldrich); 50 nM Lysotracker (Life Technologies) was used for lysosome staining; 0.1 μg/mL LD540 neutral lipid dye (kindly provided by Christoph Thiele, LIMES, Bonn) was used for the staining of lipid droplets as previously described [66].

### Immunofluorescence microscopy

Immunofluorescent staining was performed on paraformaldehyde-fixed cells as described earlier [15]. Images were taken with Zeiss Axioplan II fluorescence microscope equipped with an AxioCam MRm camera (Zeiss, Jena, Germany) and processed with Axiovision 4.6 software (Zeiss). Co-localization analysis was performed by visual inspection of coded slides.

Live cell imaging was performed in μ-Slide I chambers (Ibidi, Martinsried, Germany). Approximately 60,000 cells were seeded in each chamber. After 24 hours, cells were transfected with 0.5 μg DNA and 1.5 μL X-tremeGENE 9 and incubated for 24 hours. Imaging was performed using Axiovert 200 M microscope with an AxioCam MRM camera (Zeiss) fitted with a wrap-around temperature-controlled chamber.

### SDS-PAGE and Western Blot

Approximately 100,000 MEFs/well were seeded in 12-well cell culture plates. Samples were induced with 200 U/mL IFN-γ (PeproTech, Rocky Hill, NJ) for 24 hours, with 40 μg/ml RAP (Sigma-Aldrich) for 2 hours, or left untreated. Samples were lysed in 80 μL of modified RIPA buffer (0.1 % NP-40 and 1 % SDS) by 5 minutes of shaking and 5 minutes of 95 °C incubation. The amounts of protein were quantified with BCA assay (Pierce, Rockford, IL, USA) and 20 μg of samples were subjected to 10 % SDS-PAGE and western blot analysis. Membranes were blocked in 5 % non-fat dry milk in TBST buffer and probed for the proteins of interest with the indicated primary and secondary antibodies. The intensity of the bands was quantified by Quantity One software (Biorad, Hercules, CA, USA).

### Cell death assay

Approximately 200,000 immortalized MEFs or 300,000 BMDMs were seeded on a 6-cm cell culture dish in 2 mL of medium. Samples were induced with 200 U/mL IFN-γ, 10 mM LeuLeuOMe (Sigma-Aldrich), 500 ng/mL lipopolysaccharide (Sigma-Aldrich), or left untreated for 24, 48, or 72 hours. Before analysis, 500 μL of medium containing 2.5 μg/mL PI (Sigma-Aldrich) and 2.5 μg/mL Hoechst 33342 (Sigma-Aldrich) dye was added directly to the cells without removing the old medium and incubated for 15 minutes at 37 °C. Ten images of each sample were taken with the fluorescent Axiovert 200 microscope (Zeiss). The number of Hoechst-positive nuclei (blue) and PI-positive (red) punctae was quantified with the Volocity 6.3 software (Perkin Elmer, Waltham, MA) in at least 5000 cells per sample.

### DQ-BSA assay

Approximately 60,000 WT, *Irgm1*^*−/−*^, *Irgm3*^*−/−*^, and *Irgm1/Irgm3*^*−/−*^ MEFs were seeded in μ-Slide I chambers. After 24 hours, cells were induced with 200 U/mL IFN-γ or left untreated. After a further 24 hours, cells were incubated with 10 μg/mL DQ Red BSA (Life technologies) [52] and in parallel treated with 200 U/mL IFN-γ or 200 nM Bafilomycin A1 (Sigma-Aldrich) or left untreated. After 1 hour, samples were twice washed with PBS and further incubated in medium containing the corresponding concentration of IFN-γ and Bafilomycin A1. After 3 hours, pictures of live cells on blinded slides were taken and the number of DQ-positive punctae was manually quantified.

### Cathepsin B release analysis

To establish a concentration of LeuLeuOMe that can induce LMP without killing the cell, *Irgm1*^−/−^ MEFs were treated with different concentrations of this reagent for 24 hours. Cell death was measured with PI/Hoechst staining assay. The highest tested concentration of LeuLeuOMe that did not strongly affect cell survival was 3.75 mM (Additional file 9: Figure S9A). It was further microscopically tested whether the degree of LMP is correlated to the number of cathepsin B punctae. To induce LMP, *Irgm1*^*−/−*^ MEFs were treated with 2, 3, or 4 mM of LeuLeuOMe or left untreated for 24 hours. Cells were fixed and stained with the anti-cathepsin B antibody. Pictures of the samples were taken and cathepsin B punctae in the cells were manually blind counted (Additional file 9: Figure S9B). Samples treated with higher concentrations of LeuLeuOMe had fewer cathepsin B punctae per cell, indicating that cathepsin B staining corresponds to the extent of the LMP in a dose-dependent manner.

### Cooperativity and hierarchy of IRG loading to lysosomes

*Irgm1*^*−/−*^ cells were induced with IFN-γ and stained for LAMP1 and combinations of different IRG proteins (Fig. 3c). The number of lysosomes that co-localize with only one or both IRG proteins was quantified in 20–30 cells per sample (~20–80 lysosomes per cell).

Probabilities that the observed values arise from independent loading of the two IRG proteins tested were based on a χ^2^ comparison of observed values with expectation from independent loading.

## Additional files

Additional file 1: Figure S1. Activated Irga6 co-localizes with the lysosomes. There is no cross-reactivity between 10D7 and 1D4B antibodies. *Irgm1* ^−/−^ mouse embryonic fibroblasts were induced with 200 U/mL IFN-γ for 24 hours. Cells were fixed and stained with mouse antibody 10D7, which stains the Irga6 in GTP-bound state, and rat antibody 1D4B, which stains LAMP1 lysosomal marker. The antibodies were visualized with secondary antibodies Alexa 488 donkey-anti-mouse and Alexa 555 goat-anti-rat. To test the cross-reactivity, one of the primary or secondary reagents was omitted in each sample. The images of stained cells were taken. Representative microscopic images of GTP-bound Irga6 and LAMP1 are shown, with omitted antibody being annotated. (TIF 14983 kb) Additional file 2: Figure S2. Irga6 co-localizes with LAMP1 in GMS transfected cells. Gene Switch 3T3 cells stably transfected with inducible Irga6 were stimulated with mifepristone and simultaneously transiently transfected with combinations of pGW1H-Irgm1, pGW1H-Irgm2, and pGW1H-Irgm3. Upon 24 hours of induction, samples were fixed and stained as in Fig. 2a. Representative microscopic images of Irga6 and lysosome co-localization are shown. Arrows point at the Irga6 structures magnified at the end of each panel in the following array: upper left: Irga6, upper right: LAMP1, lower left: overlay, lower right: phase contrast. (TIF 10553 kb) Additional file 3: Figure S3. Irgd and Irgb10 co-localize with LAMP1 in *Irgm1* ^−/−^ mouse embryonic fibroblasts (MEFs). *Irgm1* ^−/−^, *Irgm3* ^−/−^, and *Irgm1/Irgm3* ^−/−^ MEFs were induced with 200 U/mL IFN-γ for 24 hours. Cells were fixed and stained with anti-Irgd antiserum (**A**) or anti-Irgb10 antiserum (**B**) and anti-LAMP1 antibody. Representative microscopic images of Irgd and LAMP1 (**A**) or Irgb10 and LAMP1 (**B**) co-localization are shown. Arrows point at the Irgd or Irgb10 structures magnified at the end of each panel in the following array: upper left: Irgd or Irgb10, upper right: LAMP1, lower left: merge, lower right: phase contrast. Scale bars represent 10 μM. (TIF 7689 kb) Additional file 4: Figure S4. Irga6 does not co-localize with mitochondria in GMS-deficient cells. **A** gs3T3 cells stably transfected with inducible *Irga6* were stimulated with mifepristone for 24 hours. Samples were fixed and stained with anti-Irga6 antibody (10D7) and anti-Tom20 antibody. Representative microscopic images of Irga6 and Tom20 co-localization are shown. Arrows point at the Irga6 structures magnified at the end of each panel in the following array: upper left: Irga6, upper right: Tom20, lower left: merge, lower right: phase contrast. Scale bars represent 10 μM. **B** Quantification of S4A, showing percent of Irga6 structures co-localizing with Tom20. 50 cells per sample were quantified. (TIF 5787 kb) Additional file 5: Figure S5. Irga6 does not co-localize with the Golgi in GMS-deficient cells. **A** Wild type, *Irgm1* ^−/−^, *Irgm3* ^−/−^, and *Irgm1/Irgm3* ^−/−^ mouse embryonic fibroblasts were induced with 200 U/mL IFN-γ for 24 hours. Cells were fixed and stained for Irga6 (165/3) and for Golgi marker GM130. Representative microscopic images of Irga6 and Golgi co-localization are shown. Arrows point at the Irga6 structures magnified at the end of each panel in the following array: upper left: Irga6, upper right: GM130, lower left: merge. Scale bars represent 10 μM. **B** Quantification of S5A, showing percent of Irga6 structures co-localizing with GM130. 50 cells per sample were quantified. (TIF 8669 kb) Additional file 6: Figure S6. Irga6 does not co-localize with Golgi in GMS-transfected cells. **A** gs3T3 cells stably transfected with inducible *Irga6* were stimulated with mifepristone and simultaneously transiently transfected with different combinations of pGW1H-Irgm1, pGW1H-Irgm2, and pGW1H-Irgm3 for 24 hours. Samples were fixed and stained for Irga6 and GM130. Representative microscopic images of Irga6 and Golgi co-localization are shown. Arrows point at the Irga6 structures magnified at the end of each panel in the following array: upper left: Irga6, upper right: Golgi, lower left: merge, lower right: phase contrast. **B** Quantification of S6A, showing percent of Irga6 structures co-localizing with GM130. 50 cells per sample were quantified. (TIF 13762 kb) Additional file 7: Figure S7. Number of autophagosomes is increased in IFN-γ-induced *Irgm1* ^*−/−*^ cells. **A** Wild type and *Irgm1* ^−/−^ mouse embryonic fibroblasts were induced with 200 U/mL IFN-γ for 24 hours and/or 40 μg/mL rapamycin for 2 hours, or left untreated. Cells were fixed and stained for LC3. Representative microscopic images of LC3 punctae and phase contrast are shown. Scale bars represent 10 μm. **B** Quantification of S7A, showing average number of LC3 punctae per cell. 50 cells per sample were counted and the results of two independent experiments are shown. (TIF 7475 kb) Additional file 8: Figure S8 LC3 co-localizes with LAMP1 in IFN-γ-induced *Irgm1* ^−/−^ cells. Wild type (WT) and *Irgm1* ^−/−^ mouse embryonic fibroblasts (MEFs) were induced with 200 U/mL IFN-γ for 24 hours, 40 μg/mL rapamycin for 2 hours, or left untreated. Cells were fixed and stained for LC3 and LAMP1. Representative microscopic images of LC3 and LAMP1 localization in WT and *Irgm1* ^−/−^ MEFs are shown. Arrows point at the LC3 structures magnified at the end of each panel in the following array: upper left: LC3, upper right: LAMP1, lower left: merge, lower right: phase contrast. Scale bars represent 10 μM. (TIF 9874 kb) Additional file 9: Figure S9. Cathepsin B punctae quantification in *Irgm1* ^−/−^ mouse embryonic fibroblasts (MEFs). **A** *Irgm1* ^−/−^ MEFs were treated with different concentrations of lysosomal permeabilization agent LeuLeuOMe or left untreated for 24 hours. Cells were stained with propidium iodide (PI) and Hoechst dye and analyzed as in Fig. 8. Percentage of PI-positive cells is shown; 5000–10000 cells per sample were quantified. **B** *Irgm1* ^−/−^ MEFs were treated with 2, 3, or 4 mM of LeuLeuOMe or left untreated for 24 hours. Cells were fixed and stained with anti-cathepsin B antibody AF965. Average number of cathepsin B-positive punctae per cell is shown; 50 cells per sample were blind counted. **C** WT MEFs and *Irgm1* ^*−/−*^ MEFs were treated with 200 U/mL IFN-γ, 3 mM LeuLeuOMe, or left un-treated for 24 hours. Cells were fixed and stained with anti-cathepsin B antibody. Representative images of cathepsin B staining and phase contrast are shown. **D** Quantification of S9C, showing mean number of cathepsin B punctae per cell; 100 cells per sample were blind counted and the results of two independent experiments are shown. (TIF 19085 kb)
