# Supplementary figures and images for: Loss of the interferon-γ-inducible regulatory immunity-related GTPase (IRG), Irgm1, causes activation of effector IRG proteins on lysosomes, damaging lysosomal function and predicting the dramatic susceptibility of Irgm1-deficient mice to infection
Source: BMC Biol. 2016 Apr 20;14:33. doi: 10.1186/s12915-016-0255-4 (PMC4837601; doi:10.1186/s12915-016-0255-4)

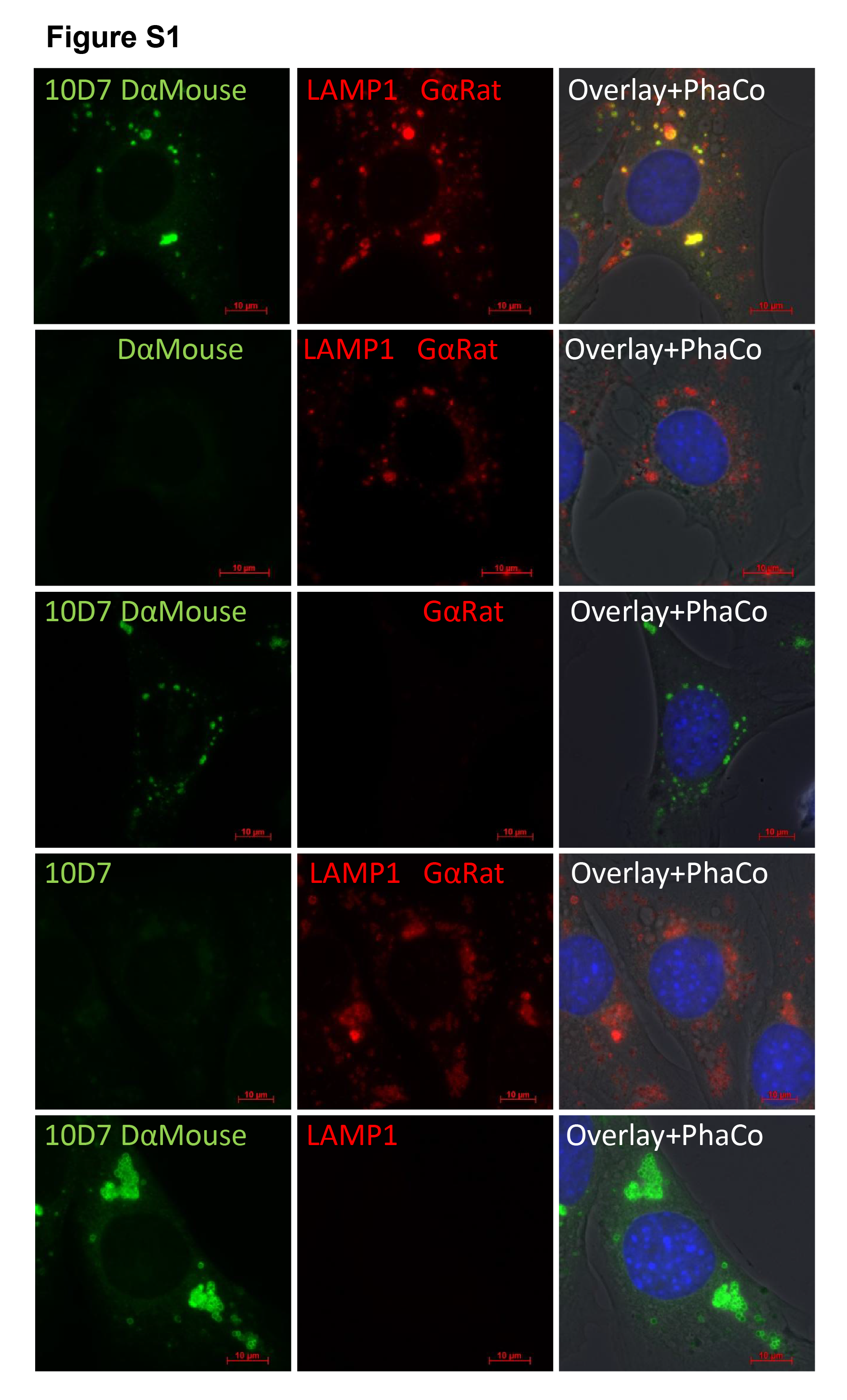

Supplement: Additional file 1: Figure S1. — Activated Irga6 co-localizes with the lysosomes. There is no cross-reactivity between 10D7 and 1D4B antibodies. Irgm1 −/− mouse embryonic fibroblasts were induced with 200 U/mL IFN-γ for 24 hours. Cells were fixed and stained with mouse antibody 10D7, which stains the Irga6 in GTP-bound state, and rat antibody 1D4B, which stains LAMP1 lysosomal marker. The antibodies were visualized with secondary antibodies Alexa 488 donkey-anti-mouse and Alexa 555 goat-anti-rat. To test the cross-reactivity, one of the primary or secondary reagents was omitted in each sample. The images of stained cells were taken. Representative microscopic images of GTP-bound Irga6 and LAMP1 are shown, with omitted antibody being annotated. (TIF 14983 kb) [file 12915_2016_255_MOESM1_ESM.tif]

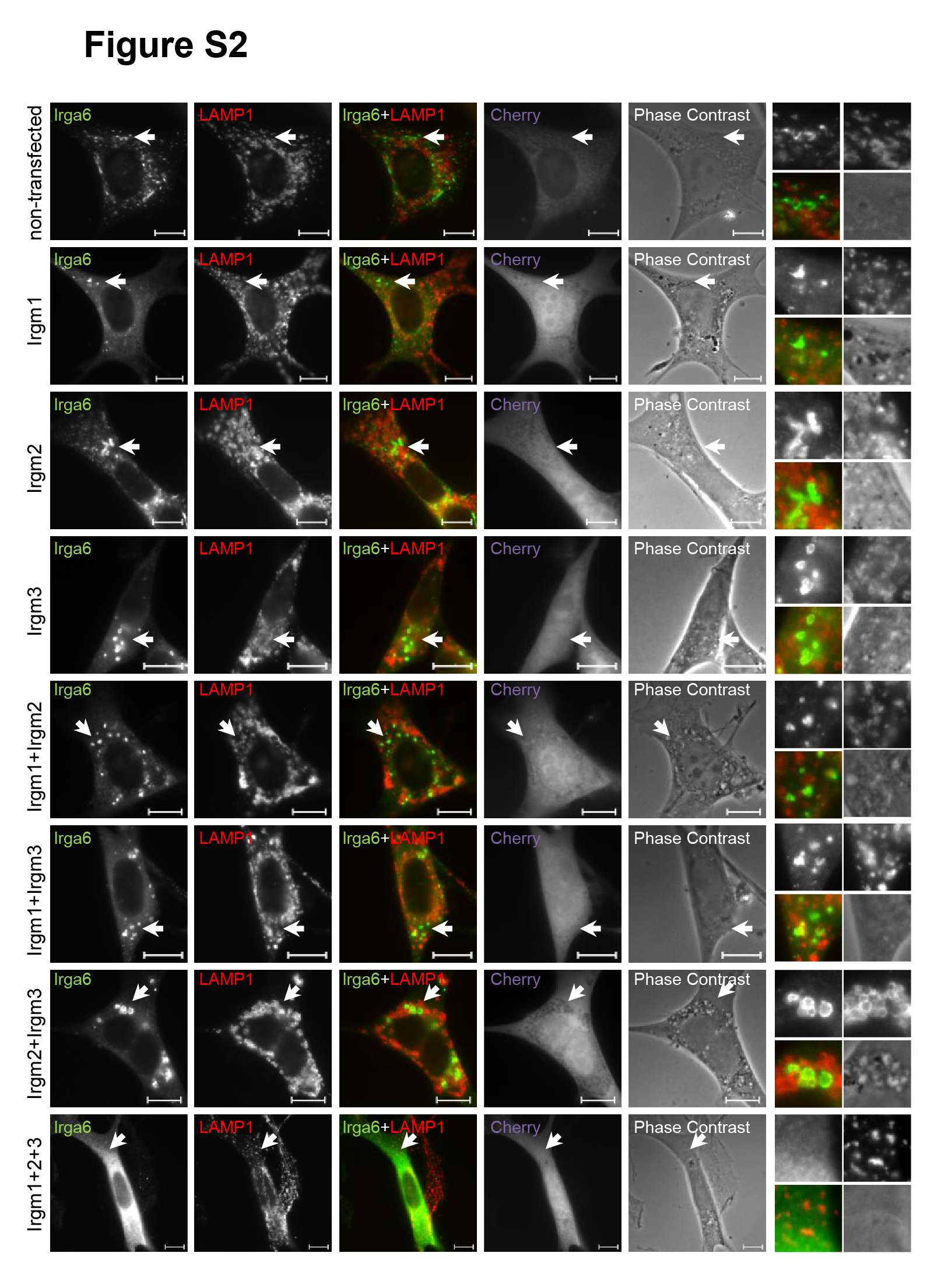

Supplement: Additional file 2: Figure S2. — Irga6 co-localizes with LAMP1 in GMS transfected cells. Gene Switch 3T3 cells stably transfected with inducible Irga6 were stimulated with mifepristone and simultaneously transiently transfected with combinations of pGW1H-Irgm1, pGW1H-Irgm2, and pGW1H-Irgm3. Upon 24 hours of induction, samples were fixed and stained as in Fig. 2a. Representative microscopic images of Irga6 and lysosome co-localization are shown. Arrows point at the Irga6 structures magnified at the end of each panel in the following array: upper left: Irga6, upper right: LAMP1, lower left: overlay, lower right: phase contrast. (TIF 10553 kb) [file 12915_2016_255_MOESM2_ESM.tif]

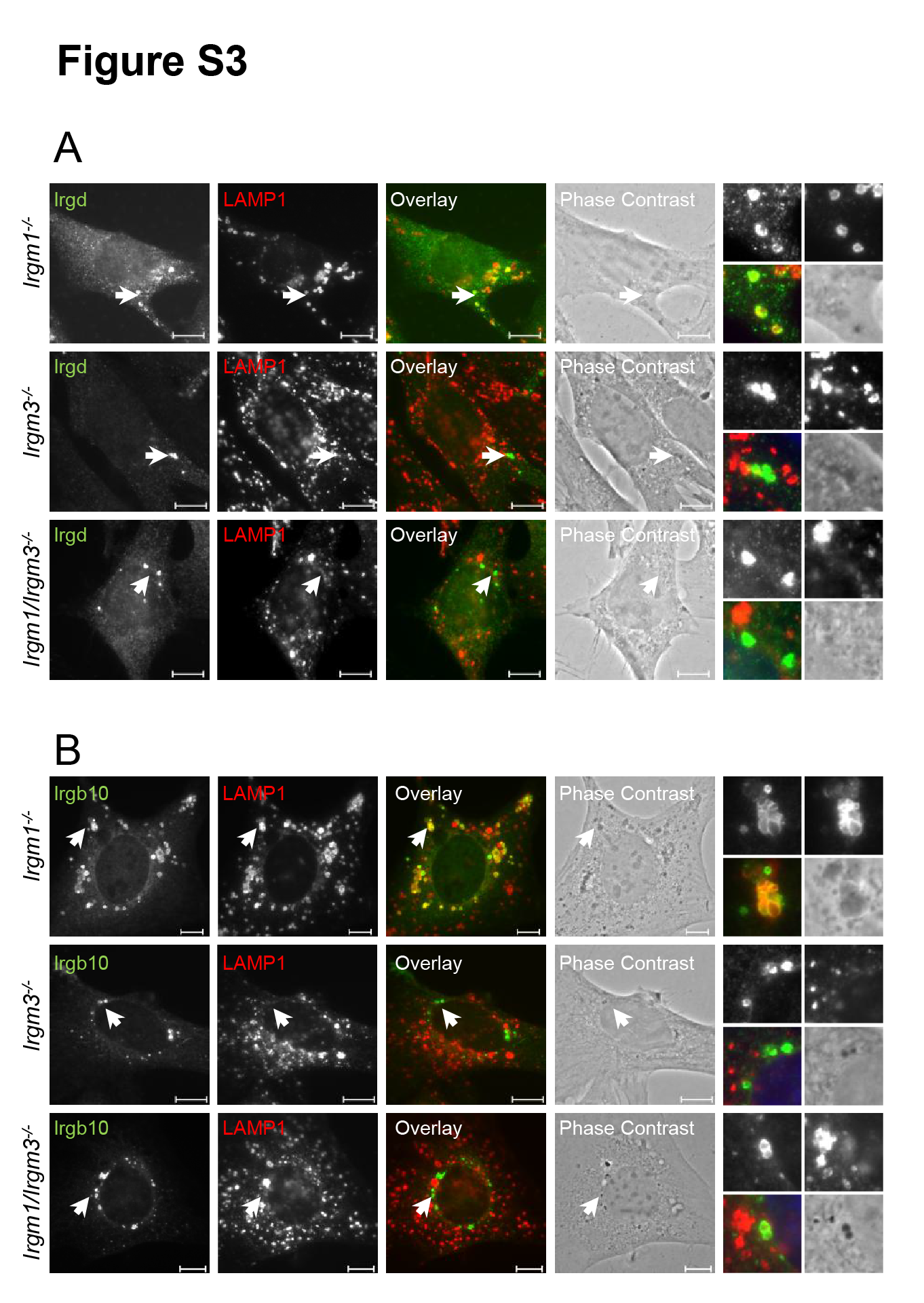

Supplement: Additional file 3: Figure S3. — Irgd and Irgb10 co-localize with LAMP1 in Irgm1 −/− mouse embryonic fibroblasts (MEFs). Irgm1 −/−, Irgm3 −/−, and Irgm1/Irgm3 −/− MEFs were induced with 200 U/mL IFN-γ for 24 hours. Cells were fixed and stained with anti-Irgd antiserum (A) or anti-Irgb10 antiserum (B) and anti-LAMP1 antibody. Representative microscopic images of Irgd and LAMP1 (A) or Irgb10 and LAMP1 (B) co-localization are shown. Arrows point at the Irgd or Irgb10 structures magnified at the end of each panel in the following array: upper left: Irgd or Irgb10, upper right: LAMP1, lower left: merge, lower right: phase contrast. Scale bars represent 10 μM. (TIF 7689 kb) [file 12915_2016_255_MOESM3_ESM.tif]

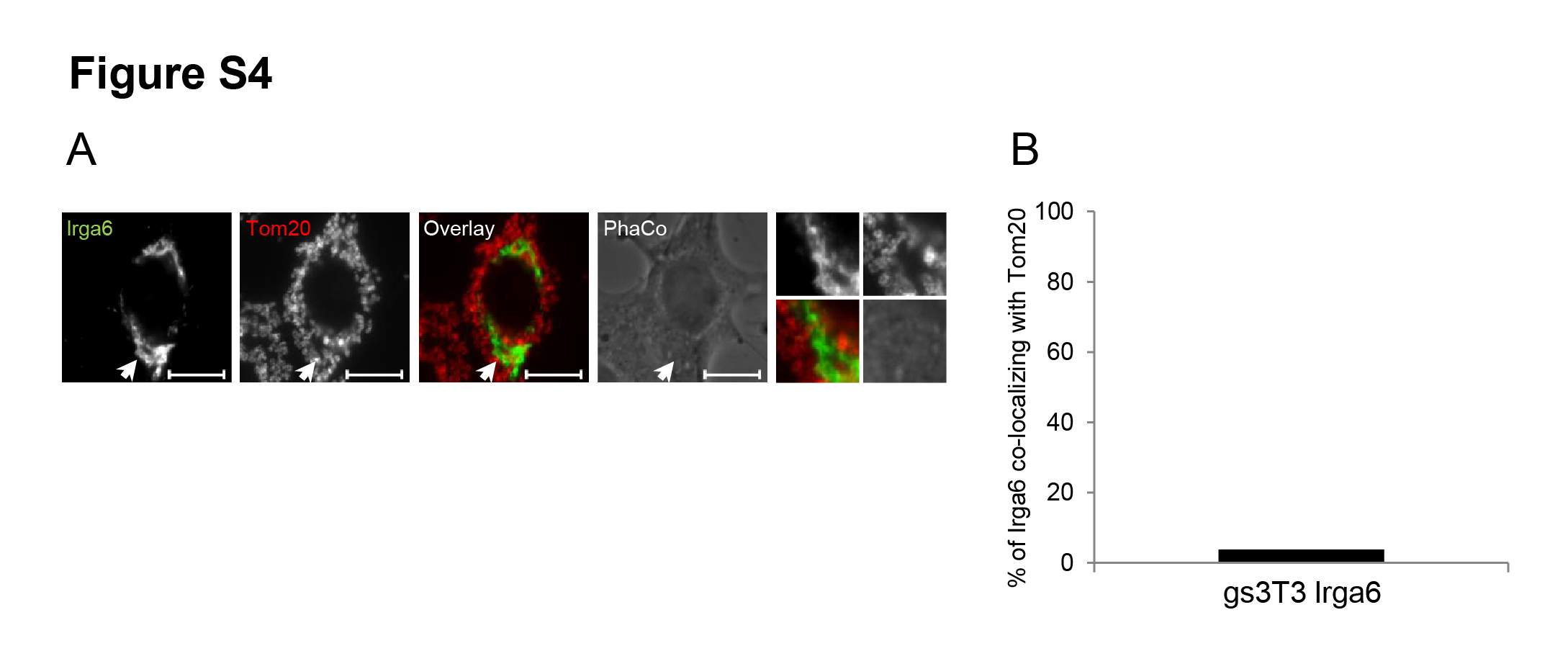

Supplement: Additional file 4: Figure S4. — Irga6 does not co-localize with mitochondria in GMS-deficient cells. A gs3T3 cells stably transfected with inducible Irga6 were stimulated with mifepristone for 24 hours. Samples were fixed and stained with anti-Irga6 antibody (10D7) and anti-Tom20 antibody. Representative microscopic images of Irga6 and Tom20 co-localization are shown. Arrows point at the Irga6 structures magnified at the end of each panel in the following array: upper left: Irga6, upper right: Tom20, lower left: merge, lower right: phase contrast. Scale bars represent 10 μM. B Quantification of S4A, showing percent of Irga6 structures co-localizing with Tom20. 50 cells per sample were quantified. (TIF 5787 kb) [file 12915_2016_255_MOESM4_ESM.tif]

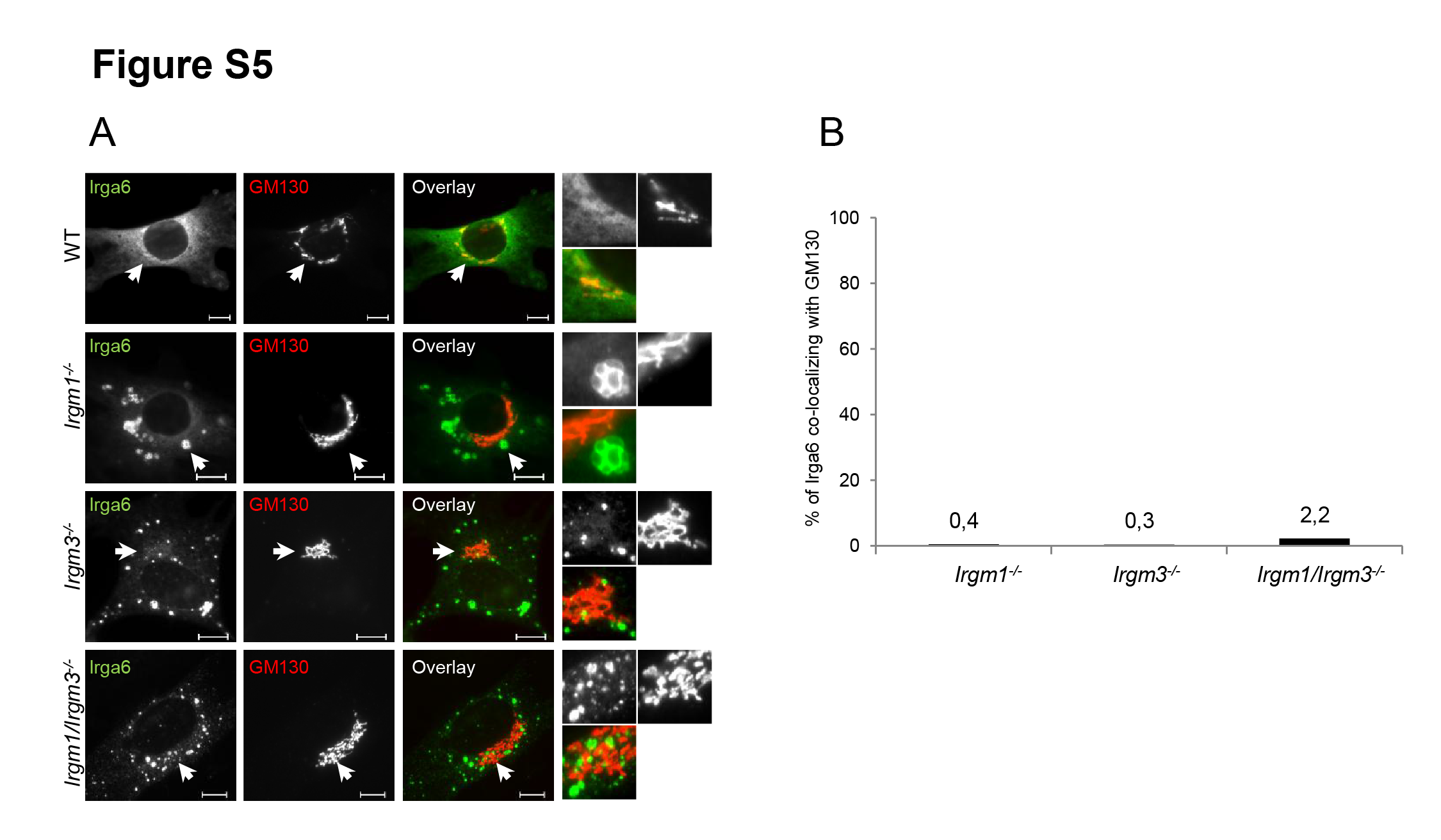

Supplement: Additional file 5: Figure S5. — Irga6 does not co-localize with the Golgi in GMS-deficient cells. A Wild type, Irgm1 −/−, Irgm3 −/−, and Irgm1/Irgm3 −/− mouse embryonic fibroblasts were induced with 200 U/mL IFN-γ for 24 hours. Cells were fixed and stained for Irga6 (165/3) and for Golgi marker GM130. Representative microscopic images of Irga6 and Golgi co-localization are shown. Arrows point at the Irga6 structures magnified at the end of each panel in the following array: upper left: Irga6, upper right: GM130, lower left: merge. Scale bars represent 10 μM. B Quantification of S5A, showing percent of Irga6 structures co-localizing with GM130. 50 cells per sample were quantified. (TIF 8669 kb) [file 12915_2016_255_MOESM5_ESM.tif]

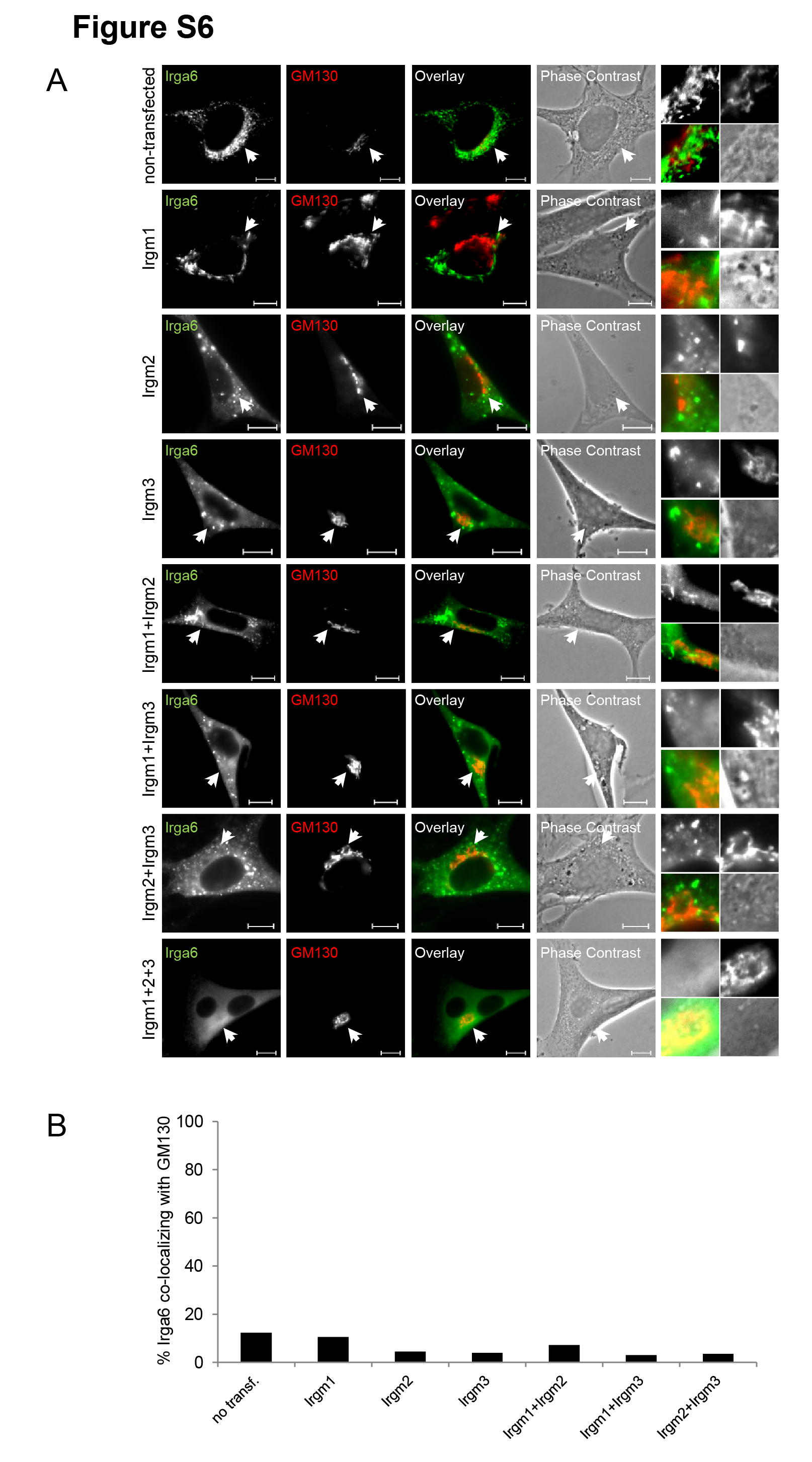

Supplement: Additional file 6: Figure S6. — Irga6 does not co-localize with Golgi in GMS-transfected cells. A gs3T3 cells stably transfected with inducible Irga6 were stimulated with mifepristone and simultaneously transiently transfected with different combinations of pGW1H-Irgm1, pGW1H-Irgm2, and pGW1H-Irgm3 for 24 hours. Samples were fixed and stained for Irga6 and GM130. Representative microscopic images of Irga6 and Golgi co-localization are shown. Arrows point at the Irga6 structures magnified at the end of each panel in the following array: upper left: Irga6, upper right: Golgi, lower left: merge, lower right: phase contrast. B Quantification of S6A, showing percent of Irga6 structures co-localizing with GM130. 50 cells per sample were quantified. (TIF 13762 kb) [file 12915_2016_255_MOESM6_ESM.tif]

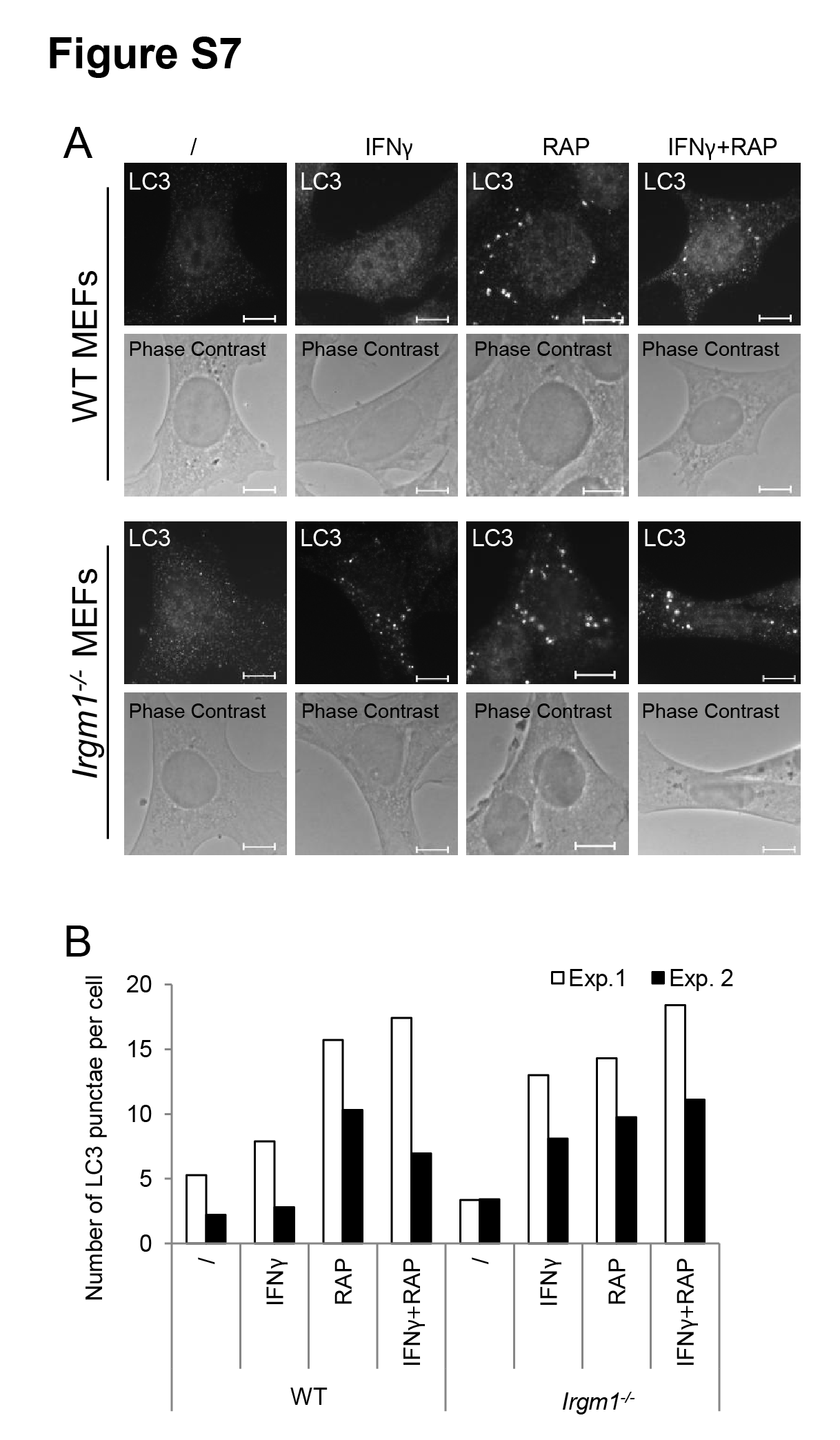

Supplement: Additional file 7: Figure S7. — Number of autophagosomes is increased in IFN-γ-induced Irgm1 −/− cells. A Wild type and Irgm1 −/− mouse embryonic fibroblasts were induced with 200 U/mL IFN-γ for 24 hours and/or 40 μg/mL rapamycin for 2 hours, or left untreated. Cells were fixed and stained for LC3. Representative microscopic images of LC3 punctae and phase contrast are shown. Scale bars represent 10 μm. B Quantification of S7A, showing average number of LC3 punctae per cell. 50 cells per sample were counted and the results of two independent experiments are shown. (TIF 7475 kb) [file 12915_2016_255_MOESM7_ESM.tif]

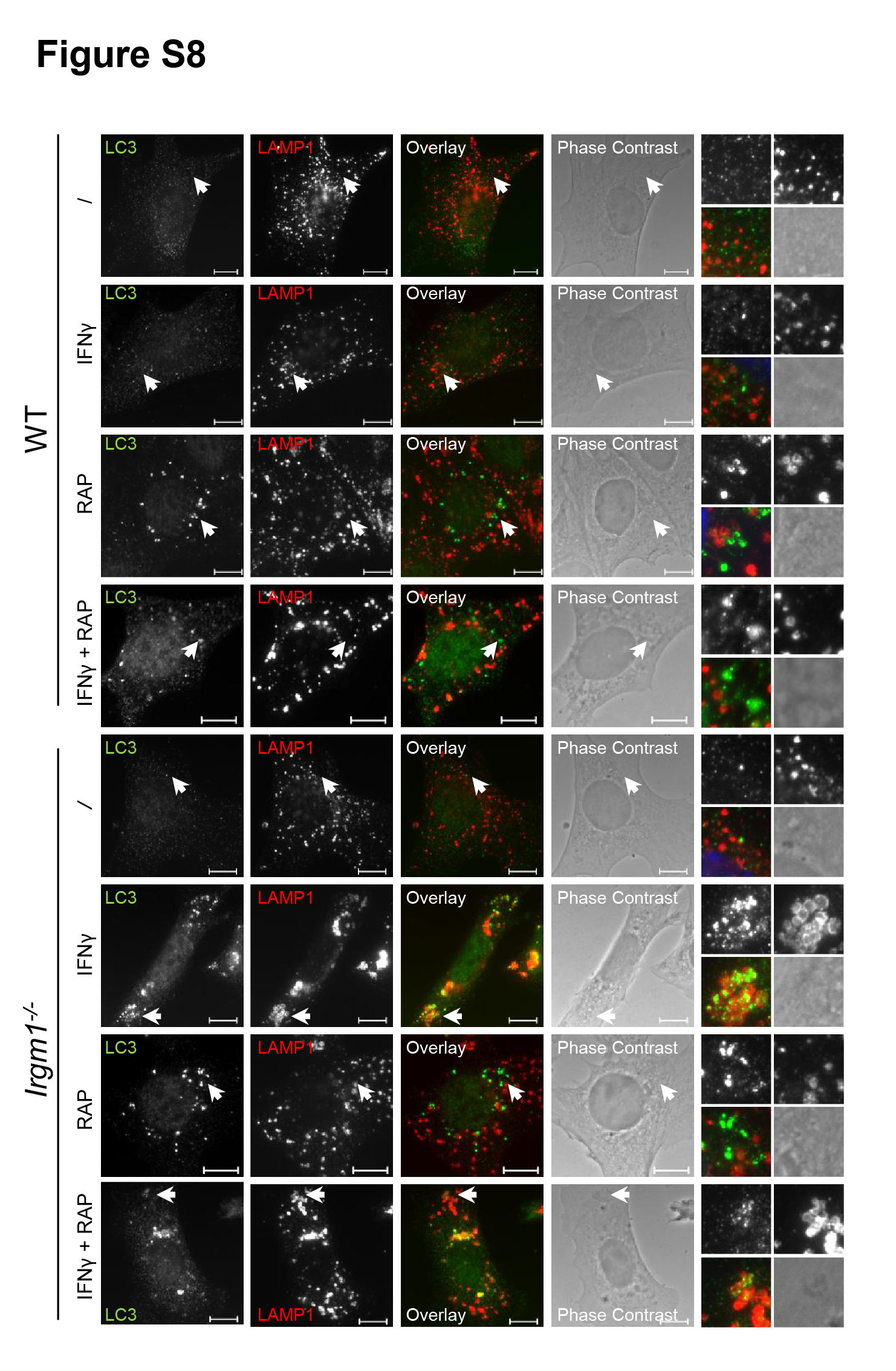

Supplement: Additional file 8: Figure S8 — LC3 co-localizes with LAMP1 in IFN-γ-induced Irgm1 −/− cells. Wild type (WT) and Irgm1 −/− mouse embryonic fibroblasts (MEFs) were induced with 200 U/mL IFN-γ for 24 hours, 40 μg/mL rapamycin for 2 hours, or left untreated. Cells were fixed and stained for LC3 and LAMP1. Representative microscopic images of LC3 and LAMP1 localization in WT and Irgm1 −/− MEFs are shown. Arrows point at the LC3 structures magnified at the end of each panel in the following array: upper left: LC3, upper right: LAMP1, lower left: merge, lower right: phase contrast. Scale bars represent 10 μM. (TIF 9874 kb) [file 12915_2016_255_MOESM8_ESM.tif]

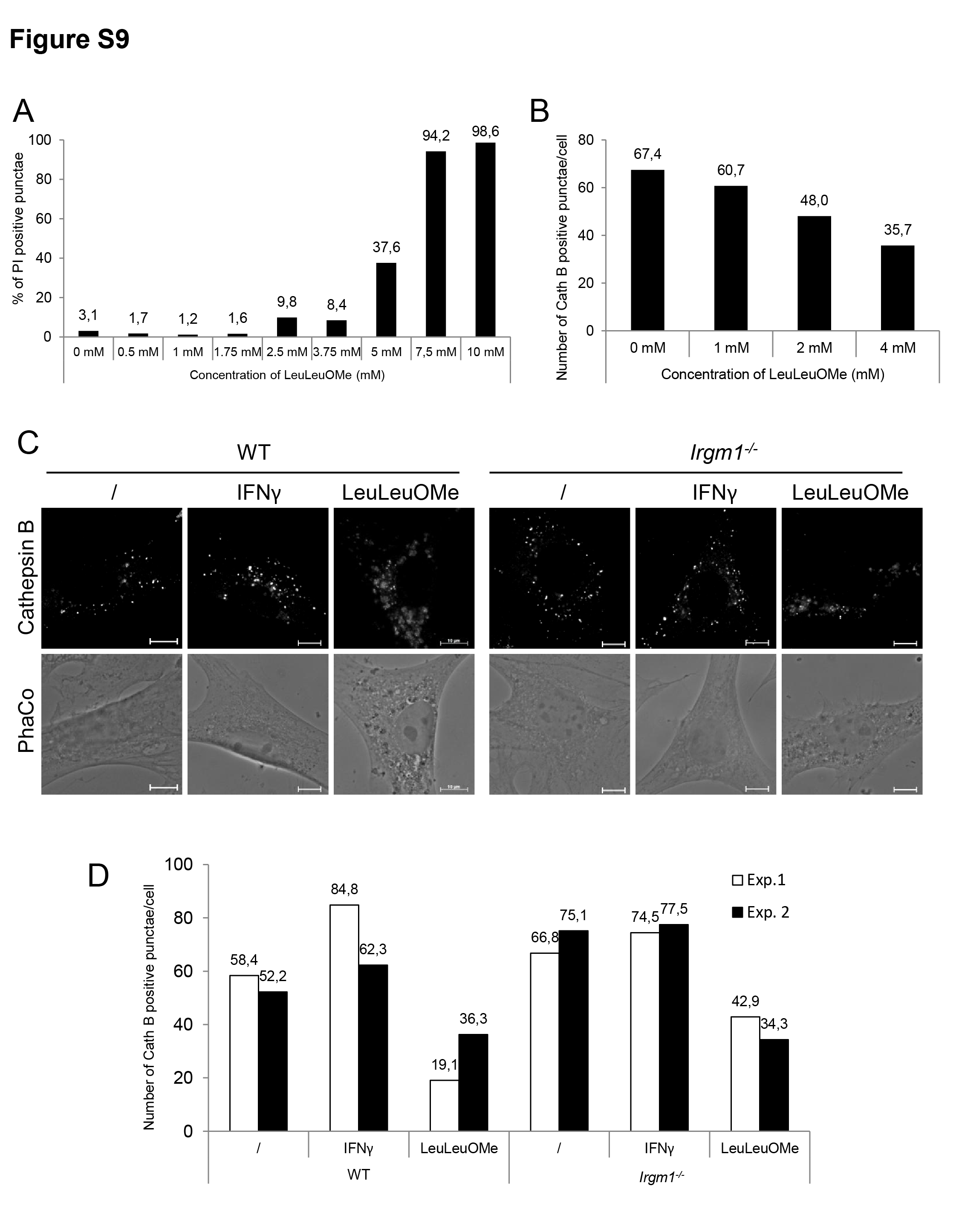

Supplement: Additional file 9: Figure S9. — Cathepsin B punctae quantification in Irgm1 −/− mouse embryonic fibroblasts (MEFs). A Irgm1 −/− MEFs were treated with different concentrations of lysosomal permeabilization agent LeuLeuOMe or left untreated for 24 hours. Cells were stained with propidium iodide (PI) and Hoechst dye and analyzed as in Fig. 8. Percentage of PI-positive cells is shown; 5000–10000 cells per sample were quantified. B Irgm1 −/− MEFs were treated with 2, 3, or 4 mM of LeuLeuOMe or left untreated for 24 hours. Cells were fixed and stained with anti-cathepsin B antibody AF965. Average number of cathepsin B-positive punctae per cell is shown; 50 cells per sample were blind counted. C WT MEFs and Irgm1 −/− MEFs were treated with 200 U/mL IFN-γ, 3 mM LeuLeuOMe, or left un-treated for 24 hours. Cells were fixed and stained with anti-cathepsin B antibody. Representative images of cathepsin B staining and phase contrast are shown. D Quantification of S9C, showing mean number of cathepsin B punctae per cell; 100 cells per sample were blind counted and the results of two independent experiments are shown. (TIF 19085 kb) [file 12915_2016_255_MOESM9_ESM.tif]
